# Forest soil carbon is threatened by intensive biomass harvesting

**DOI:** 10.1038/srep15991

**Published:** 2015-11-04

**Authors:** David L. Achat, Mathieu Fortin, Guy Landmann, Bruno Ringeval, Laurent Augusto

**Affiliations:** 1 INRA, Bordeaux Sciences Agro, UMR 1391 ISPA, 33140 Villenave d’Ornon, France; 2 AgroParisTech, UMR 1092 LERFoB, 54000 Nancy, France; 3 INRA, Centre de Nancy-Lorraine, UMR 1092 LERFoB, 54280 Champenoux, France; 4 ECOFOR, 42 rue Scheffer, F-75116 Paris, France

## Abstract

Forests play a key role in the carbon cycle as they store huge quantities of organic carbon, most of which is stored in soils, with a smaller part being held in vegetation. While the carbon storage capacity of forests is influenced by forestry, the long-term impacts of forest managers’ decisions on soil organic carbon (SOC) remain unclear. Using a meta-analysis approach, we showed that conventional biomass harvests preserved the SOC of forests, unlike intensive harvests where logging residues were harvested to produce fuelwood. Conventional harvests caused a decrease in carbon storage in the forest floor, but when the whole soil profile was taken into account, we found that this loss in the forest floor was compensated by an accumulation of SOC in deeper soil layers. Conversely, we found that intensive harvests led to SOC losses in all layers of forest soils. We assessed the potential impact of intensive harvests on the carbon budget, focusing on managed European forests. Estimated carbon losses from forest soils suggested that intensive biomass harvests could constitute an important source of carbon transfer from forests to the atmosphere (142–497 Tg-C), partly neutralizing the role of a carbon sink played by forest soils.

Forests contain more carbon than the atmosphere^1^,^2^,^3^ and, as such, are a major component of the carbon cycle on Earth. Compared with other terrestrial ecosystems, forests store some of the largest quantities of carbon per surface area of land^4^. As a result, the carbon storage capacity of land could be improved through afforestation, or decreased by deforestation^4^,^5^. While such land-use changes have well-known consequences on land carbon, the long-term impact of forest managers’ decisions remains unclear relative to the global carbon cycle, and strategies regarding carbon by management of forests are conflicting^6^. One school of thought proposes that forests should be allowed to accumulate carbon in the long-term because old-growth forests are active carbon sinks^7^. An alternative approach proposes an intensification of wood harvesting to replace fossil carbon in the production of manufactured objects and energy^2^. The best strategy for managing forest carbon as a means of mitigating climate change is still a controversial issue^1^. Indeed, while collecting more biomass can help in the substitution of fossil energy by fuelwood, it also results in the reduction of carbon stocks sequestered in trees^8^, and in turn, a possible reduction in the future rate of carbon accumulation, due to the removal of the largest trees which have the highest accumulation rates^9^. Furthermore, although it has been established that forest management can modify stocks of soil organic carbon (SOC)^10^, the extent to which the intensity and frequency of biomass harvests might be deleterious to forest SOC remains unclear because of the difficulty in monitoring this compartment of the ecosystem accurately^6^,^10^, and due to the high number of factors involved^11^. The complexity of this question has led to many uncertainties^1^ and inconclusive debates^12^,^13^,^14^.

Here we report a global assessment of the consequences of different management practices on soil organic carbon storage in forests. We focused on soils because they are generally the largest carbon pools of forest ecosystems^15^, are less exposed to climatic extremes than trees^16^, and because little is known about their responses to changes in management or the environment^2^,^10^. The assemblage of results published on this topic in peer-reviewed journals yielded large databases comprising experimental forest sites distributed worldwide. In each forest, different practices of biomass harvest were tested, and their consequences on the soil carbon pool were monitored. We quantified the effects on SOC of the three main strategies in terms of carbon management: i) *carbon sequestration in forests*, based on unharvested forests, ii) *conventional harvests* of tree stems, used in most managed forests, and iii) *intensive harvests*, based on the collection of tree stems and logging residues (stumps, branches, foliage, and sometimes forest floor racking) to produce fuelwood^2^,^17^. Collection of trees –both in conventional and intensive harvests– can be incomplete or total. Thus, we additionally took into account if conventional, or intensive, harvests were carried out during a thinning (the felling and logging of a proportion of trees to promote the growth of the residual trees^18^) or a clear-cutting (the felling and logging of all trees, followed by seedling planting, sowing, or natural forest regeneration^19^). Because in practice most intensive harvests were done at clear-cutting, we studied possible differences between thinning and clear-cutting for conventional harvests only.

We compiled data from 284 forest sites and built two datasets related to conventional harvests and intensive harvests, respectively (see Methods). Although the majority of these forests are located in the Northern hemisphere, in North America and Europe, under temperate or cold climates (Tables S1 and S2 in Supplementary Information), they are distributed worldwide, representing all types of managed forests (Fig. 1; Fig. S1 in Supplementary Information). The consolidated datasets included a total of 2,028 values of SOC change in different soil layers up to 135 years after biomass harvesting (Figs S2 and S3). Soil layers were grouped into four classes depending on their depth (the organic layer above the mineral soil profile: forest floor “F”; top, mid and deep mineral soil layers: “T”, “M”, and “D”). Cumulated soil layers were also examined (“TM”, “TMD”, “FT”, “FTM” and “FTMD”).

## Results

### Conventional harvests

The impact assessment of conventional harvests, as compared with unharvested forests (first dataset), indicated that around 22% of SOC in the F layer was lost due to harvesting operations (Fig. 2A; Fig. S4A). This loss of carbon in forest floors appeared to be long lasting as it was still clearly apparent a decade after harvesting (Fig. 3A) and possibly required more than half a century to be fully compensated (Fig. 4 and Fig. S2). Surprisingly, there were only slight differences between thinning and clear-cutting (Fig. S4A), except during the first decade when there were higher SOC losses after clear-cutting than after thinning (Fig. 3A and Fig. S2). During the first decade, SOC losses also tended to increase with increasing thinning intensity (Fig. S5). There was, however, no thinning frequency effect or forest age effect.

The response of SOC stocks in the upper mineral layer was clearly different from that of the forest floor. In the T layer, SOC stocks often remained stable (Figs 2A and 3B). However, carbon losses did occur in some cases, especially when this topsoil layer was disturbed as a result of forest clear-cutting with heavy machinery, or *soil preparation* before seedling plantation (Fig. S6A).

Despite an overall non-significant change of carbon storage in the T layer, conventional harvests reduced the carbon stock of the “FT” upper soil by 14% on average as a result of the important loss in the forest floor (Fig. 2A; Fig. S4A). This general decrease of SOC in the upper part of the soil profile (i.e. F+T) was compensated by an accumulation beneath (Fig. 2A; Fig. S4A): when deep layers (D) and above all medium layers (M) were taken into account, the balance of SOC losses *versus* SOC gains was not significantly different from zero (the mean value for the complete FTMD soil profile = −6% SOC).

### Intensive harvests

The results obtained from our second dataset indicated that intensive harvests strongly reduced SOC stocks in woody debris (WD) and in the F layer, relative to stem-only harvests (Fig. 2B; Fig. S4B). In addition, the entire mineral soil (TMD) was also negatively impacted (Fig. 2B), especially when the forest floor was racked and exported from the forest (Fig. S4B). Unfortunately, published studies containing information for the complete organic plus mineral soil profile (i.e. FTMD) were scarce. This gap in the literature prevented us from directly assessing the effect of intensive harvests on SOC stocks in forests. Nevertheless, because both the organic soil layers (WD and F) and the mineral soil profiles (TMD) showed a clear decrease, a general reduction of the soil carbon stock was likely to occur. After one decade, SOC losses were no longer detected in the topsoil (T), although they were still reported in the F layer (Fig. 3; Fig. S3). There were negative relationships between SOC losses in the F layer and SOC losses in mineral soils (r^2^ = 0.42–0.61), suggesting transfers of carbon from the forest floor to mineral layers. But, these possible vertical fluxes appeared to be of small magnitude and as such, they could not compensate for SOC losses from mineral soil layers (Fig. 2B), at least during the decade following intensive harvest.

Firstly, we showed that, compared with unharvested forests, conventional harvests of forest biomass had a moderate impact on SOC stocks (Fig. 2A; see *conventional harvests* subsection, above). Here, we found that, contrary to conventional harvests, intensive harvests of forest biomass had a negative impact on SOC stocks (Fig. 2B). At this stage, we tried to test the effects of intensive harvests compared with unharvested forests. However, because published data comparing intensive harvests with unharvested forests are scarce, we were unable to perform statistical tests directly. Instead, we combined our two datasets: *i)* intensive harvests *versus* conventional harvests, and *ii)* conventional harvests *versus* no harvests, using a bootstrap resampling analysis (see Supplementary Methods). Our simulations indicated that intensive harvests were able to induce large SOC losses in comparison with untouched forests (Fig. 2C). SOC losses occurred mainly in the forest floor (−37%) and in deep soil layers (−7%).

### Simulation at the European scale

Managed forests in Europe correspond to approximately 142 million hectares with 38% in boreal regions and 62% in temperate regions. Assuming mean SOC stocks in the whole organic plus mineral soil profile to be 277 and 95 Mg-C ha^−1^ respectively for boreal and temperate forests, total SOC stocks in European managed forests represent 23.5 Pg-C (15.1 and 8.4 Pg-C in boreal and temperate forests, respectively). Using the SOC distribution within the soil profile and percentage losses due to intensive harvesting under boreal and temperate climates we obtained in this study, we estimated that the implementation of management strategies based on intensive harvests would cause a loss of organic carbon in forest soils, ranging between 142 and 497 Tg-C, depending on the scenario of management conversion (see Methods). We calculated a mean annual SOC loss over three decades, because in the present study the impacts of intensive harvests have been assessed over a period of 30 years (Fig. S3). Thus, we estimated that the mean annual loss of soil organic carbon in European forests could be between 5 to 17 Tg-C year^−1^.

## Discussion

### Conventional harvests

Our results, showing a negative effect in the F layer and little overall impact in the T layer, were in accordance with previous findings^19^,^20^,^21^ and suggested a negative impact of conventional harvests on forest SOC stocks. Nevertheless, this conclusion is based solely on the most superficial part of soils and investigating the influence of conventional harvests on deeper soil layers led to different conclusions. Our study showed an accumulation of SOC in the M layer which resulted in a net increase of SOC storage in the combined soil layers (TMD; Fig. S4A). When considering the whole soil profile (FTMD), the SOC gain in the mineral layers compensated for the SOC loss observed in the forest floor (Fig. 2A; Fig. S4A). Overall, conventional biomass harvests had no, or only a slightly negative but statistically non-significant, impact on carbon in forest soils when deeper soil layers were also taken into account. This result brings a different perspective than the usual conclusion of decreased SOC stocks when only considering shallow horizons, as usually done in many case studies of the literature.

Our observations on the dynamics of carbon stocks in forest soils following conventional harvests can be explained by several processes. In forests, leaf and wood litterfall is, at best, quantitatively low during the first few years following the removal of standing trees. This reduced flux of organic carbon from aboveground tree biomass to the forest floor has a negative effect on the forest floor stocks^18^,^20^,^22^,^23^. Subsequently, as trees grow, litterfall production increases and enables the recovery of carbon stocks in the forest floor^18^,^20^. Besides the changes in litterfall production, an increase in organic matter decomposition is also expected to occur and to negatively impact SOC storage. Decomposition rates generally increase in the superficial part of soils immediately after harvests due to soil disturbance and changes in microclimatic conditions (increased solar radiation and, thereby, soil temperature) until canopy closure^21^,^23^,^24^. The accumulation of SOC we observed in the M layer was probably due to the inputs of carbon from dead roots immediately following harvesting^25^, combined with the migration of dissolved organic carbon from the soil layers above^26^. In addition, in sites where foresters *prepare* the soil before planting (e.g. by soil ploughing), soil disturbance can mix the different soil layers; the forest floor and some logging debris being typically incorporated into the mineral soil^27^. These results demonstrated that, contrary to widely held opinion, conventional harvests have no globally negative impact on organic carbon stocks of forest soils.

### Intensive harvests

Then, we investigated the extent to which intensifying biomass harvests by exporting the logging residues, to supply fuelwood chains for instance^2^, can change the pattern observed with conventional harvests. Similarly to conventional harvests, intensive harvests induced large SOC losses in the F layer. Large SOC losses in the F layer seemed to reduce SOC losses in mineral soils (see negative relationships in Fig. S7D and E), possibly due to the migration of dissolved organic carbon from forest floor decomposition^26^ or the mixing of soil layers due to soil preparation^27^. However, at best, this input from the F layer yielded some compensation, but it never reached the stage of SOC accumulation in the M and D layers (see negative relationships between SOC losses in F and in mineral soil layers). It implies that, contrary to conventional harvests, there was usually no complete compensation between organic and mineral soil layers under intensive harvests and the overall impact of intensive removal of forest biomass on SOC stock remained negative. This impact was even more negative when intensive harvests were compared with unharvested forests, such as those of the *old-growth* strategy.

### Heterogeneity of SOC response – environmental factors implied

It is worth stressing that the average effects of forestry practices reported above masked large regional and local disparities. For instance, not all forests showed an accumulation of organic matter in the mid-part of their soil after conventional harvests. This high variability was visible also in topsoils and deep soil layers, but was coherent for a given soil profile because the responses of mid and deep soil layers were influenced by topsoil layer behaviour: when SOC loss or gain was observed in the topsoil, a SOC change in the same direction was generally recorded in deeper soil (Fig. S7A-C). As for conventional harvests, high inter-site variability existed after intensive harvest, but was logical with concomitant losses or stabilities of SOC between mineral soil layers (Fig. S7F).

Such inter-site heterogeneity can be explained by climatic gradients and ecosystem characteristics. In accordance with studies reporting the impact of deforestation^5^,^28^, SOC losses in topsoils due to conventional harvests increased with increasing initial SOC (Fig. S8A and B), the latter being itself partly controlled by climate (Fig. S8C). However, climate was a poor predictor of SOC dynamics after conventional harvests, with no significant difference when comparing tropical, temperate, and boreal forests (*P* > 0.1), perhaps due to insufficient data for tropical forests (Table S1). Climatic influence was clearer for intensive harvests, as demonstrated by the positive relationships between SOC losses and mean annual temperature and evapotranspiration (Fig. 5). Carbon losses were consequently lower under cold climates compared with temperate climates (Fig. 6; not enough data for tropical climates, see Table S1). We interpreted this pattern to be a consequence of soil microclimatic conditions induced by forest management. Indeed, less logging residues were left on site after intensive harvests, leading to microclimatic changes such as an increase in soil temperature in spring and summer due to the role of the debris in regulating temperature variations^29^,^30^. Sites affected by intensive harvests were probably exposed to larger increases in soil temperature in temperate regions than in cold regions, which in turn could lead to higher increases in SOC decomposition in temperate regions^5^,^30^. There were not enough sites in the dataset to assess the effect of intensive harvests on SOC under tropical climates (Table S1), but high temperatures in these regions were expected to favour larger increases in soil temperature and consequently higher organic matter decomposition and SOC losses, as observed in temperate regions^5^. This expectation was in line with a recent study which demonstrated a strong relationship between the carbon turnover time and climate in terrestrial ecosystems^31^.

Soil type was another factor modifying SOC response to biomass harvests. For instance, highly weathered soils had an accumulation of SOC in their topsoil layer after a conventional harvest (Fig. S6B). Finally, forest composition seemed significantly influencing our results. As already reported in the literature^19^, a comparison of hardwood forests with coniferous and mixed forests suggests that the former experience higher SOC losses than the latter. However, as hardwoods and conifers are not equally distributed along global climatic gradients, we tested a possible climatic bias. In practice, we repeated the comparison between these groups of tree species, but using a subset of our data for which mean annual temperature and precipitation were in the same range of values for all forests. Under this analytical restriction, the influence of vegetation composition was not significant, which suggested that the observed effect of forest composition might be related to climate. Similarly, no significant effect of forest age on SOC change could be detected in our datasets.

### Simulation at the European scale—Conclusion

The aggregation of results collected from experimental forests indicated that intensive harvests have unwarranted consequences on soil carbon stocks and, consequently, could have an impact on carbon budgets. To quantify this possible effect, we extrapolated the development of intensive harvests in the European Union under different scenarios of intensive forestry development. Our simulations indicated a total loss of 5–17 Tg-C year^−1^, depending on the scenario. We recognize that these estimates are broad extrapolations which require further investigation, by using process-based modelling for instance. On the other hand, they provided pertinent indications in comparison with other processes involved in the carbon cycle. Indeed, Luyssaert and his colleagues^32^ calculated that the carbon sink of European forest soils was around 29 Tg-C year^−1^. In terms of magnitude this value was comparable to our estimates of annual SOC losses from the same region. In other words, changing to more intensive harvests would have detrimental consequences, because soils would fix less carbon due to the loss of part of this sink, as shown by our results. Under our most severe scenario (i.e. 17 Tg-C year^−1^), approximately 57% of the soil carbon sink was offset by unintended losses.

Our findings clearly demonstrate that using the *intensive harvest* strategy at its maximum level decreases soil carbon storage. Besides SOC losses, the removal of logging residues has other negative effects on forest soils, such as a decrease in nutrient availability (mainly due to increased exportation of nutrients) which could lead to a reduction in site fertility^2^,^33^,^34^,^35^ and tree growth^34^,^35^, thereby reducing carbon storage in tree biomass in the long term^12^. In sites where inherent soil fertility is low, intensive harvests should consequently be discouraged, to prevent productivity decline from occurring. Otherwise, the negative effects of intensive harvests should be mitigated by reducing the removal rate of logging residues^2^,^17^,^34^,^35^ and preserving the forest floor^35^.

Because the carbon budget also depends on carbon sequestration in standing trees^8^ and on the substitution of fossil carbon by biomass^2^, the question of whether additional harvesting of forest biomass has a positive impact on the greenhouse gas balance remains an open debate^12^. Conversely, our study provided accurate estimates of the losses of soil organic carbon that should be taken into account when assessing the potential benefits of forest bioenergy on the global carbon budget.

## Methods

### Meta-analysis compilation of data at the stand scale

Our global analysis was based on observations collected from 238 peer-reviewed publications. Gathering all these studies, we built two datasets. The first included values of organic C storage in soils under the influence of *conventional harvests* (i.e. treatment = tree stem harvest *versus* control = no harvest^18^,^19^; N = 118 and 80 sites for forest clear-cutting and forest thinning, respectively; N = 1462 values of soil organic carbon (SOC) changes, considering all soil layers, treatments and sampling dates for each site). As clear-cutting involves more severe disturbance than thinning, we systematically searched for possible differences between these two types of biomass export. Nevertheless, because there was generally no difference, clear-cutting and thinning were often merged in the results.

The second dataset encompassed the effects of *intensive harvests* (i.e. whole-tree harvest treatment = harvest of logging residues (e.g. branches, foliage, or stumps) in addition to stem harvest *versus* control = stem-only harvest^34^; N = 86 sites; N = 566 values of SOC changes, considering all soil layers, treatments and sampling dates). Most of data about intensive harvests were at clear-cutting stage.

**Figure 1:**
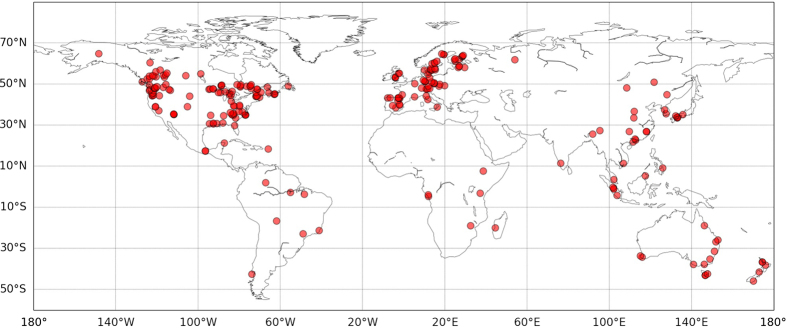
Distribution of the sites used in this meta-analysis on the effects of conventional and intensive harvests on soil C stocks. See more details on the geographical location of the sites in Fig. S1. Map created in Python Language version 2.7 (Python Software Foundation; www.python.org), using the *basemap* package (https://pypi.python.org/pypi/basemap/1.0.7) of the *matplotlib* library (http://matplotlib.org).

**Figure 2:**
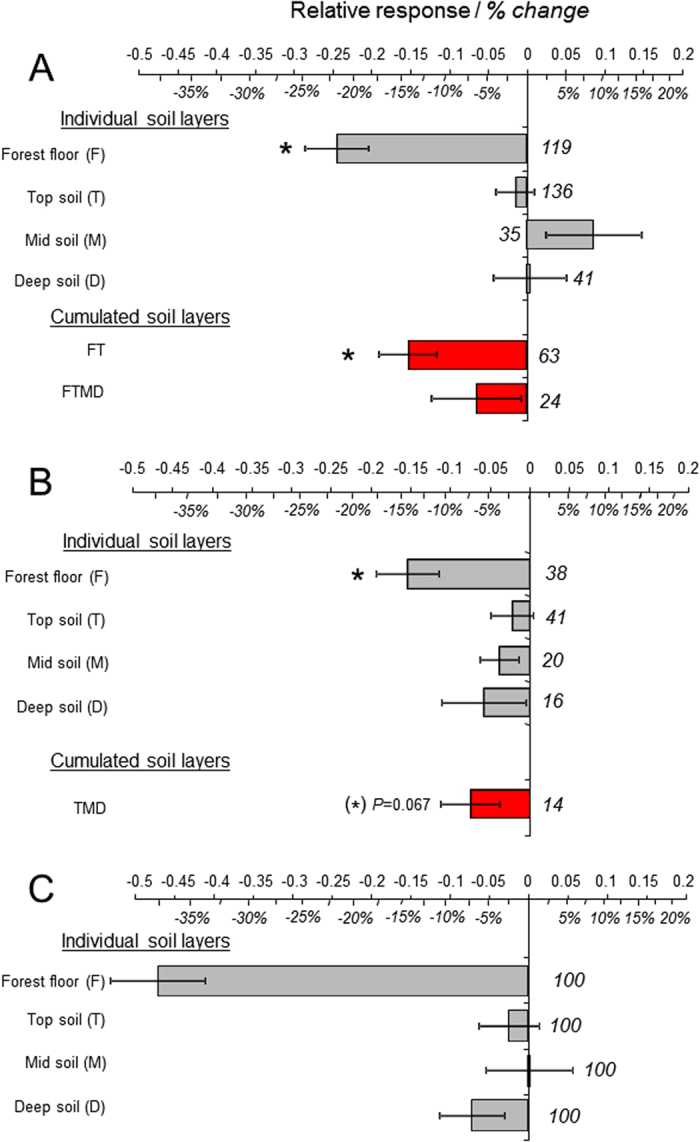
General effects of conventional and intensive harvests on SOC stocks as a function of soil depth (individual soil layers) and in the entire soil profile (cumulated soil layers). (**A**) Effects of conventional harvest (clear-cutting and thinning; means ± standard errors). (**B**) Effects of intensive harvest compared with stem-only harvest (means ± standard errors). (**C**) Combined effects of conventional and intensive harvests. Values are expressed as relative responses: (**A**) log(clear-cutting or thinning harvest/unharvested control) (**B**) log(whole-tree harvest/stem-only harvest) (**C**) log(whole-tree harvest/unharvested control). For the sake of clarity, comparisons between treatments and controls are also presented as the mean arithmetic difference (in italics, expressed in %). Results in (**C**) were obtained using the two datasets (data in **A**,**B**) and a bootstrap resampling method. For each panel, number of case studies (or sites) and number of bootstrap samples are shown in italics to the right of each bar. There were not enough data for FTMD in (**B**). Significant differences between relative responses and 0 are denoted by an asterisk (t test). See more results in Fig. S4.

**Figure 3:**
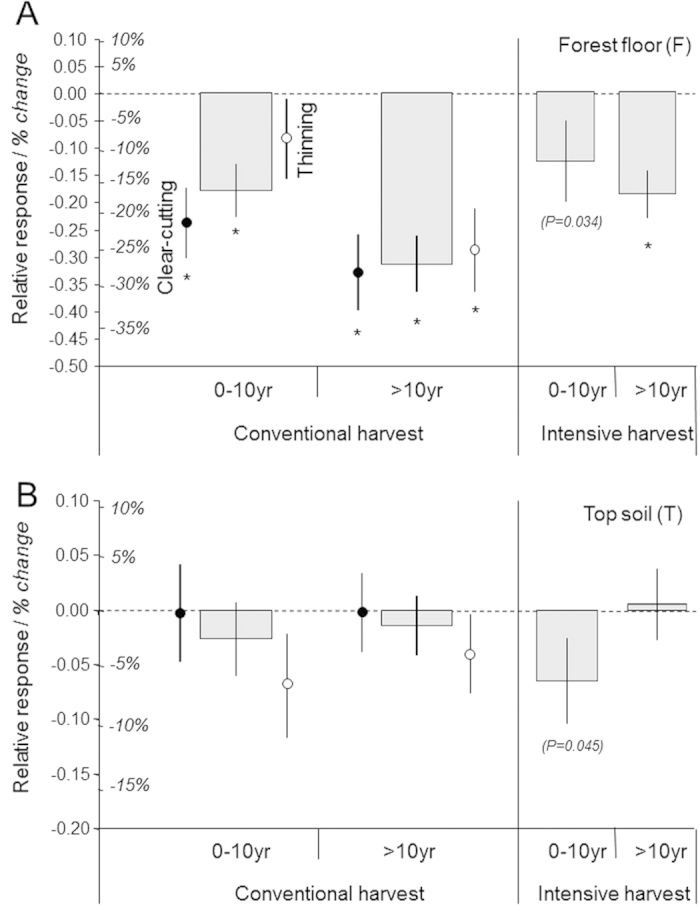
Effects of conventional and intensive harvests on SOC stocks in forest floor (F) and top mineral soil (T) in relation to time elapsed since harvesting. (**A**) Forest floor. (**B**) Top soil. Effects were assessed considering two periods (0–10 years and > 10 years since harvesting; Means ± standard errors). Values are expressed as relative responses: log(clear-cutting or thinning harvest/unharvested control) or log(whole-tree harvest/stem-only harvest). For the sake of clarity, comparisons between treatments and controls are also presented as the mean arithmetic difference (in italics, expressed in %). Number of case studies (or sites) ranged from 16 to 100. Significant differences between relative responses and value 0 are denoted by an asterisk (*t* test). The *P* values in brackets were calculated using all intensive harvest treatments (whole-tree harvest and whole-tree + forest floor harvest). Effects of conventional clear-cutting on C stocks in the forest floor are shown for more time classes in Fig. 4.

**Figure 4:**
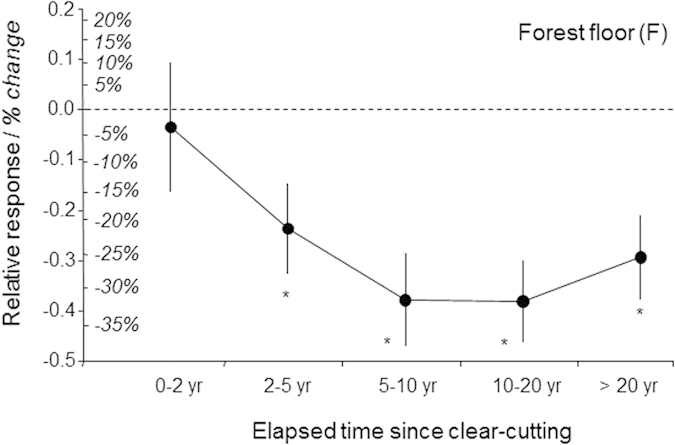
Effects of conventional clear-cutting harvest on SOC stocks in forest floor (F) in relation to time elapsed since harvesting. Effects were assessed considering five periods (0–2, 2–5, 5–10, 10–20 and >20 years since harvesting; Means ± standard errors). Values are expressed as relative responses: log(conventional clear-cutting harvest/unharvested control). For the sake of clarity, comparisons between treatments and controls are also presented as the mean arithmetic difference (in %). Number of case studies (or sites) ranged from 12 to 31. Significant differences between relative responses and value 0 are denoted by an asterisk (*t* test). Temporal changes associated with other harvest types are shown in Supplementary Information (conventional harvest at thinning: Fig. S2; intensive harvest: Fig. S3).

**Figure 5:**
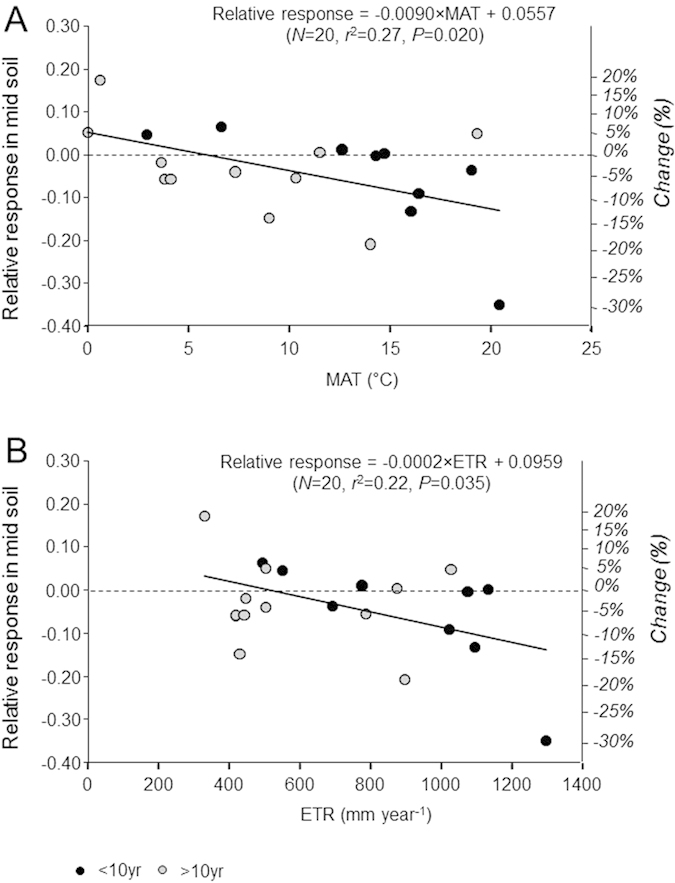
Effects of intensive harvest on C stocks in mid soil (M) related to mean annual temperature (MAT) and effective evapotranspiration (ETR). (**A**) MAT; (**B**) ETR. Values are expressed as relative responses [log(whole-tree harvest/stem-only harvest)]. For the sake of clarity, comparisons between treatments and controls are also presented as the mean arithmetic difference (% higher or lower). A similar trend (*P* < 0.1) was observed between ETR and SOC losses for the topsoil layer also (data not shown).

Sites were distributed worldwide (Fig. 1 and Fig. S1), but most of them were located in the Northern hemisphere under temperate or cold climates (Tables S1 and S2). We collected SOC data, sampling depth and explanatory variables including geographical location, altitude, time since harvesting, thinning intensity, soil disturbance (i.e. ploughing after clear-cutting and before planting), vegetation, climate, and soil type. To assess the consequences of forest management practices on SOC storage as a function of soil depth and in the entire soil profile, SOC data were classified into four soil layers (see Supplementary Information for more details): forest floor (F: organic soil layer above the mineral soil profile), top mineral soil (T: mean sampling depth ≤10 cm), mid soil (M: 11–20 cm) and deep soil (D: >20 cm). SOC stocks (in Mg-C ha^−1^) were subsequently calculated in each soil layer, in the mineral soil profile (e.g. TMD = T + M + D) and in the organic plus mineral soil profile (e.g. FT = F + T, FTMD = F + T + M + D).

We assessed the magnitude of changes in SOC stocks in response to conventional harvests and intensive harvests using the concept of *effect size* and a calculation of the relative response [log(treatment/control)] in each soil layer or in the soil profile. For the sake of clarity, comparisons between treatments and controls were also presented as the mean arithmetic difference or percentage change (higher or lower). To quantify the effect of intensive harvests as compared with unharvested controls, we combined the two datasets and used a bootstrap resampling method (see Supplementary Methods).

First, we evaluated the general effects of biomass harvest on SOC storage. To test the significance of the effect of each treatment (conventional or intensive harvests) on SOC stocks, the relative response was compared to 0 using a *t* test. Then, we explored the causes which explained the results and their heterogeneity. To do this, relationships between the relative response and explanatory variables (e.g. time elapsed since harvesting, initial SOC concentration, mean annual temperature) were assessed using either linear or non-linear regressions. Differences among classes of explanatory variables (e.g. elapsed time, soil types, climate classes) in the relative response were also assessed using one-way ANOVA.

Detailed information about the methods used in this paper is presented in the Supplementary Information.

### Simulation at the European scale

**Figure 6:**
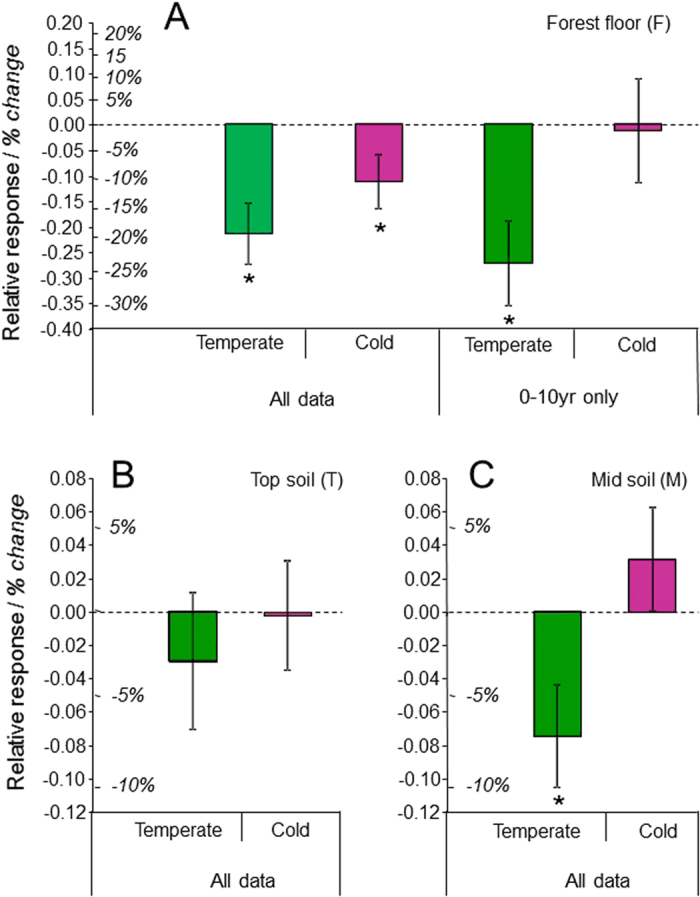
Effect of intensive harvest on C stocks in the forest floor (F), top soil (T) and mid soil (M) related to Köppen climate classes. (**A**) forest floor (all sites or selected sites (time elapsed since harvesting <10 years)); (**B**) top soil; (**C**) mid soil (all sites). Means ± standard errors. Values are expressed as relative responses [log(whole-tree harvest/stem-only harvest)]. For the sake of clarity, comparisons between treatments and controls are also presented as the mean arithmetic difference (% higher or lower). Number of sites ranged from 7 to 23 (insufficient data for tropical climates). Significant differences between relative responses and value 0 are denoted by an asterisk (*t* test). There were also significant differences among classes (ANOVA, *P* = 0.040 for the mid soil, *P* = 0.078 for the forest floor with selected sites (0–10 years)).

In a final stage, we estimated the consequences of intensive harvests in Europe. We focused on Europe because 1) a carbon budget of European forests was available^32^, 2) the great majority of those forests were managed using conventional harvesting (primary unmanaged forests correspond to only 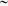
4% of total European forested area^36^), and 3) the relative importance of intensive forestry was likely to increase in upcoming decades as a result of the commitment of European countries to increase the proportion of renewable energy in their final energy consumption^2^. Because the rate of development of intensive forestry in Europe was unpredictable^2^,^17^, we tested two different scenarios assuming that 20% or 70% of European forests currently managed using conventional harvesting would become intensively managed in the next three decades. The surface areas of European forests and their distribution in boreal or temperate regions were calculated from published data^36^. Total SOC stocks in managed European forests were then calculated based on their surface areas and mean SOC stock values per hectare. We assumed that mean SOC stocks in the whole organic plus mineral soil profile were 277 and 95 Mg-C ha^−1^ for boreal and temperate forests, respectively^3^,^15^. The impact of intensive harvests was estimated by applying the mean SOC loss value found in the present study (Fig. 6). We calculated a mean annual loss of soil carbon for our two scenarios, assuming a constant rate of loss over the 30 years, because biomass harvests could have consequences over decades^37^ and because, in the present study, the impacts of intensive harvests have been assessed over a period of 30 years (Fig. S3).

## Additional Information

**How to cite this article**: Achat, D. L. *et al.* Forest soil carbon is threatened by intensive biomass harvesting. *Sci. Rep.* **5**, 15991; doi: 10.1038/srep15991 (2015).

## Supplementary Material

Supplementary Information
