# Supplementary material for: Forest soil carbon is threatened by intensive biomass harvesting
Source: Sci Rep. 2015 Nov 4;5:15991. doi: 10.1038/srep15991 (PMC4632129; doi:10.1038/srep15991)
Supplement: Supplementary Information [file srep15991-s1.pdf]

Supplementary Information

Forest soil carbon is threatened by intensive biomass harvesting

David L. Achat[1], Mathieu Fortin[2,3], Guy Landmann[4], Bruno Ringeval[1], Laurent Augusto[1*]

[1]INRA, Bordeaux Sciences Agro, UMR 1391 ISPA, 33140 Villenave d'Ornon, France.[2]AgroParisTech, UMR 1092 LERFoB, 54000 Nancy, France. [3]INRA, Centre de Nancy-Lorraine, UMR 1092 LERFoB, 54280 Champenoux, France.[4]ECOFOR, 42 rue Scheffer, F-75116 Paris, France.

*Corresponding author. E-mail: laugusto@bordeaux.inra.fr

Supplementary Methods
Supplementary References
Supplementary Figures S1 to S13
Supplementary Tables S1 to S3
References used in the meta-analysis

Supplementary Methods

Data acquisition. We used the ISI Web of Science database and holistic non-specific queries[38,39] to locate peer-reviewed publications about the impacts of conventional harvest (clear-cutting and thinning) and intensive harvest (whole-tree harvest; i.e. exports of stems and other “logging residues”) on stocks of soil organic carbon (SOC). We selected publications using keywords related to SOC in forest soils, and forest management (e.g. “carbon” or “organic matter”, “forest”, “woodland”, or “tree”, “management”, “cut”, or “thin”). Based on a series of bibliographic requests, we first identified a group of 13,560 records (Fig. S9 in the Supplementary Information). We progressively selected relevant case studies based on specific keywords, article title, and article abstract (397 publications). Finally, we used inclusion and exclusion criteria to select publications which reported relevant data (see more details on the selection protocol in Fig. S9). To be included in our datasets, the studies had to meet the following criteria: (1) clear-cutting (felling and removal of all trees) must have been followed by tree plantation or natural tree regeneration; we excluded cases of land-use changes (conversion of forests to agricultural land, grassland or agroforestry[4,5,40]), (2) sites affected by destructive practices after clear-cutting, such as slash-and-burn treatments or top soil racking[41], were also excluded, (3) thinning (or selective logging) corresponding to the cutting and removal of a proportion of trees[18] (which might include operations such as shelterwood cutting) was included, (4) combined effects of clear-cutting and thinning were excluded whenever the experimental design was not meant to separate these two effects on SOC, (5) conventional clear-cutting and conventional thinning harvests had to be compared to unharvested controls (in experimental designs) or adjacent mature stands (paired sites with similar soil conditions and vegetation)[18,19,42], (6) to assess the effect of intensive harvests, we selected studies that compared whole-tree harvest (i.e. collection of foliage, branches and sometimes, even the forest floor; all these compartments are often called “logging residues” by foresters) to stem-only harvest[34,35,43], (7) studies on the forest floor (organic soil layer above the mineral soil profile; hereafter referred to as F) had to include SOC stock data (in Mg-C ha^{-1} or in Mg-organic matter ha^{-1}) or forest floor thickness that could be used to derive C stock values, and (8) studies on mineral soils had to include SOC data (C or organic matter, stocks or concentration in g kg^{-1} soil) and sampling depth.

The latter selection stage produced a list of 238 primary articles (see references used in the meta-analysis and Figs. S10-S13). Besides the general effects of forest management on SOC storage, we also assessed the causes of SOC heterogeneity. Therefore, in addition to SOC data, we collected the following variables as possible predictors[18,19,35,38]: geographical location (latitude, longitude), altitude, time elapsed since harvesting (i.e. time elapsed since the first thinning when stands were thinned several times), site preparation (soil ploughing following clear-cutting) and soil disturbance during logging, thinning intensity (percentage of basal area - or stems - removed, number of thinnings), pre- and post-harvest vegetation (coniferous, broadleaved, mixed), age of pre-harvest trees, mean annual temperature and precipitation, and soil type. The USDA soil classification was used to group soils into 12 classes (i.e. the 12 USDA soil orders; http://www.nrcs.usda.gov). When soils were not specified in the publications, we determined the USDA soil order based on correspondences between soil classification systems[44]. In a few cases, the identification of the soil order was based on the geographical location[45], provided that the area was homogenous enough to avoid any misclassification. On the basis of preliminary verifications (comparisons between soil orders mentioned in the references and estimated soil orders based on geographical locations), we validated this geographical approach only when the estimated soil order corresponded to a Spodosol. When not provided in the publications, we estimated the main climatic variables using the geographical location of the studied forest and global maps: mean annual temperature (MAT)

and precipitation (MAP) (http://worldclim.org/bioclim), evapotranspiration (ETR; http://ntsg.umt.edu/project/mod16), and Koeppen climate classes (http://koeppen-geiger.vu-wien.ac.at/present.htm). Contrary to the USDA soil orders, preliminary verifications on two independent datasets (*conventional harvest* dataset, and *intensive harvest* dataset; see below) showed close correspondences between altitude, MAT and MAP data reported in the publications and estimated values based on geographical locations (linear regressions for altitude: slope = 0.92-1.03, r^2 = 0.83-0.87, n = 47-128; linear regressions for MAT: slope = 1.03-1.04, r^2 = 0.96-0.97, n = 55-124; linear regressions for MAP: slope = 1.01-1.05, r^2 = 0.74-0.76, n = 62-164).

Finally, we compiled data from the 238 selected publications. From these publications, 128 references reported observations on the effects of conventional clear-cutting (118 case studies), 73 on the effect of conventional thinning (80 case studies) and 71 on the effect of intensive harvest (86 case studies). Thirty four references reported the consequences of two harvesting practices (either clear-cutting/thinning or clear-cutting/intensive harvest). Studies that compared three levels of harvest (no harvest; stem-only and whole-tree harvests at clear-cutting or thinning) were scarce. Therefore, we split results into two datasets dedicated to two kinds of comparison in terms of biomass harvest: (1) conventional clear-cutting and conventional thinning harvests *versus* no harvest, and (2) whole-tree harvest (intensive harvest) *versus* stem-only harvest.

Studies were distributed worldwide (Fig.1 and Fig. S1). Nevertheless, most sites were located in the northern hemisphere (85-90%) and corresponded to the temperate and cold climate classes. Although representing a minority of our case studies (i.e. 3-15%), forests under tropical climates were also present in our datasets (Tables S1 and S2). This unbalanced distribution can be partly explained by the fact that several management practices were excluded, such as slash-and-burn treatments or land-use changes, which are common in tropical regions[4,5].

Data handling and statistics. We split our SOC values into four soil layers: forest floor (F) and three mineral soil layers, because the consequences of conventional and intensive harvests on SOC are likely to depend on soil depth. The classification of mineral soil layers was based on the sampling depth: top soil (T was defined as sampled layers with a mean soil depth ≤ 10 cm; in our "conventional harvest" database: layer thickness = 10.8 ± 0.4 cm, mean soil depth = 5.4 ± 0.2 cm; in our "intensive harvest" database: layer thickness = 10.4 ± 0.5 cm, mean soil depth = 5.5 ± 0.2 cm), mid soil (M was defined as 11-20 cm; in our "conventional harvest" dataset: layer thickness = 12.4 ± 0.7 cm, mean soil depth = 15.7 ± 0.3 cm; in our "intensive harvest" database: layer thickness = 10.4 ± 0.6 cm, mean soil depth = 15.2 ± 0.3 cm), and deep soil (D was defined as > 20 cm; in our "conventional harvest" database: layer thickness = 21.7 ± 2.7 cm, mean soil depth = 30.5 ± 1.9 cm; in our "intensive harvest" database: layer thickness = 15.9 ± 1.6 cm, mean soil depth = 29.2 ± 1.6 cm). In our databases, 82% of complete mineral soil profiles (i.e. with the three layers T, M, and D) had a profile with a vertical length between 30 and 60 cm.

It was not possible to correct basic data for depth because it would have required the analysis of several soil layers[11]. In many case studies, the number of soil layers sampled was too low to enable data correction. Instead, we applied a method adapted to heterogeneous data[46]. We defined two thin (10 cm thick) standard soil layers for the upper part of the soil profile ('top' and 'medium' layers), where the SOC changes sharply with depth[47,48]. These thin standard layers were designed to capture any large change of SOC with depth. These two standard layers, were sandwiched between a third layer for the forest floor above, and a fourth layer for deep soil below, to take deep SOC (with slight depth gradient only) into account and, more generally, to provide an accurate representation of the complete soil profile. A comparison of this

approach with a dataset built with systematically sampled soils has shown that it was a reliable method to characterize large areas of soils[46].

In addition, data classification was based on the following explanatory variables: time elapsed since harvesting (e.g. two classes: ≤ 10 years and > 10 years), site preparation (with/without soil ploughing or disturbance), soil orders (see above) and soil weathering stages[45] (highly weathered soils = spodosols + ultisols + oxisols; slightly or moderately weathered soils = alfisols + entisols + inceptisols + mollisols; unclassified in terms of soil weathering: histosols and andisols; not present in our datasets: gelisols, vertisols, and aridisols), Koeppen climate classes and vegetation type (coniferous *vs* hardwood forests).

When not explicitly presented in a reference, we calculated the SOC stock value of each soil layer (in Mg-C ha^{-1}). The calculation was based on SOC concentration (in g-C kg^{-1}), soil layer thickness (in m) and bulk density (in Mg-soil m^{-3}).

$$SOC\ stock = 10 \times SOC\ concentration \times thickness \times bulk\ density$$

For a few studies, SOC data were calculated from the organic matter or weight loss-on-ignition data, based on the assumptions that all organic matter is lost during heating and that organic matter contains 50% carbon[49]. When bulk density was not provided in a given study, it was estimated from the SOC concentration. To do this, we used a SOC-bulk density relationship that had been previously published[5] and validated with our datasets (linear regression between measured and estimated bulk density: slope = 0.96, $r^2 = 0.73$, $n = 145$).

$$Bulk\ density = e^{(0.1926 - 0.0048 \times SOC\ concentration)}$$

For a given site, we subsequently calculated SOC stocks in the mineral soil profile (T+M and T+M+D) and the organic plus mineral soil profile (F+T, F+T+M and F+T+M+D) using SOC stocks in the individual soil layers (F, T, M and D).

As in most meta-analyses, we calculated the magnitude of change in SOC stocks in response to conventional or intensive harvest using the concept of 'effect size', hereafter referred to as *relative response*[19,35,38,42,50]. The effects of conventional harvest and intensive harvest were assessed as follows:

Conventional harvest:

$$Relative\ response = log\left(\frac{(conventional\ clear\ cutting\ or\ conventional\ thinning)}{(unharvested\ control)}\right)$$

Intensive harvest:

$$Relative\ response = log[(whole\ tree\ harvest)/(stem\ only\ harvest)]$$

The *relative response* metric allows the comparison of publications across a wide range of experimental conditions[19,38]. Values close to 0 are associated with a negligible effect of the tested treatments. Negative and positive values indicate negative and positive effects, respectively. Effect sizes in meta-analyses can be weighted by study precisions, using variances and sampling sizes[51]. Here, we used an unweighted metric because variance estimates were not available in many of the studies we found. However, contrary to many previous meta-analyses, we avoided pseudo-replicates. The number of values in a given publication depends on the number of treatments[19,35] (e.g. number of levels of thinning, soil preparation after clear-cutting, intensity of residue harvesting (branches, foliage, stumps and forest floor), etc.) or the quantity

of repeated measurements (e.g. several sampling dates). Because of the large variations in the numbers of values presented in the various publications we selected, if we had assumed that each value was an independent case study, this would have led to different statistical weights and consequently would have biased the meta-analysis[38,51]. When several relative responses corresponded to one case study, we calculated a single mean value to avoid pseudoreplications[35,38] in the different classes of explanatory variables. In these cases, we based the definition of the case study on the geographical location of the studied forest.
Another issue in most meta-analyses is the publication bias[51]. This occurs when the probability of publication depends on the statistical significance, magnitude or direction of the effect and causes a bimodal distribution of the number of studies (with low frequency with low values of relative response). In these cases, we generally found unimodal distributions of the case studies in relation with the values of relative response (Figs. S10 and S11). We consequently inferred that there was no publication bias.
For the sake of clarity, comparisons between treatments and controls are also presented as the mean arithmetic difference (i.e. percent change (higher or lower)), that was calculated after back transformation:

$$Per\ cent\ change = \left[\left(e^{relative\ response} \right) - 1 \right] \times 100$$

Because only a few references enabled a direct comparison between the three levels of biomass harvest, we used an indirect approach by combining the results of our two datasets. This *combined effect* represented the impact of intensive harvest (i.e. whole-tree harvest, the exports of stems and other “logging residues” such as branches and foliage) compared with an unharvested control. In practice, for each soil layer (F, T, M and D), we generated estimated values of the effect of conventional harvest on SOC, and the effects of intensive harvest. We used a bootstrapping method to generate 100 pairs of values for each soil layer (this number of resampled values represented the order of magnitude of the number of case studies in our datasets). Because the majority of the case studies in the dataset dedicated to conventional harvest explicitly consisted of stem-only harvest, we assumed that it was the same for the other case studies, even if the stem-only harvest was not explicitly mentioned in the reference. Therefore, in each combination, resampled values were multiplied to estimate a value of the whole-tree harvest/no harvest ratio. This ratio was then log-transformed, generating 100 values of the relative response:

$$Combined\ effect = \log \left[\left(\frac{conventional\ clear\ cutting\ or\ thinning\ harvest}{unharvest\ control} \right) \times \left(\frac{whole\ tree\ harvest}{stem\ only\ harvest} \right) \right]$$

$$\Leftrightarrow Combined\ effect = \log \left(\frac{whole\ tree\ harvest}{unharvest\ control} \right)$$

To test the significance of the effect of each harvest on SOC stocks, the relative response was compared to 0 using a *t* test. Relationships between the relative response and explanatory variables (e.g. latitude, mean annual temperature) were assessed using either linear or non-linear regressions. Differences among classes of explanatory variables (e.g. soil orders, Köppen classes, site preparation, time elapsed since harvesting) in the relative response were tested using one-way ANOVA. We also assessed the effects of conventional clear-cutting and conventional thinning on forest floor C as a function of the time elapsed since harvesting, based

on Covington's function[20,52]. This function describes a curve that decreases exponentially for a period and then returns asymptotically to its initial value[20]. Initially adjusted using organic matter stock data, we adapted the function to describe the relative response or the percent change:

$$Relative\ response = log\left(\frac{a \times time^{b} \times e^{c \times time^{d}}}{100} + 1\right)$$

$$Per\ cent\ change = a \times time^{b} \times e^{c \times time^{d}}$$

where a, b, c and d were parameters to be estimated (a and c were negative, b and d were positive).

To collect the data from the publications, we used DataThief III (version 1.5) software to extract the values from figures (when not given in tables). Data analyses were performed using SAS software (SAS Institute Inc., Cary, NC, USA) and SYSTAT (version 10, Software Inc., Chicago, IL, USA) software.

Supplementary References

38 Augusto, L., Delerue, F., Gallet-Budynek, A. & Achat, D. L. Global assessment of limitation to symbiotic nitrogen fixation by phosphorus availability in terrestrial ecosystems using a meta-analysis approach. *Glob. Biogeochem. Cycle* **27**, 804-815 (2013).

39 Pullin, A. S. & Stewart, G. B. Guidelines for systematic review in conservation and environmental management. *Conserv. Biol.* **20**, 1647-1656 (2006).

40 Bárcena, T. G. *et al.* Soil carbon stock change following afforestation in Northern Europe: a meta-analysis. *Glob. Change Biol.* **20**, 2393-2405 (2014).

41 Woodward, C. L. Soil compaction and topsoil removal effects on soil properties and seedling growth in Amazonian Ecuador. *For. Ecol. Manag.* **82**, 197-209 (1996).

42 Johnson, D. W. & Curtis, P. S. Effects of forest management on soil C and N storage: meta-analysis. *For. Ecol. Manag.* **140**, 227-238 (2001).

43 Thiffault, E. *et al.* Effects of forest biomass harvesting on soil productivity in boreal and temperate forests - A review. *Environ. Rev.* **19**, 278-309 (2011).

44 Esu, I. E. Soil Characterization, Classification and Survey. (Heinemann Educational Books, 2010).

45 Yang, X., Post, W. M., Thornton, P. E. & Jain, A. The distribution of soil phosphorus for global biogeochemical modeling. *Biogeosciences* **10**, 2525-2537 (2013).

46 Augusto, L. *et al.* Is 'grey literature' a reliable source of data to characterize soils at the scale of a region? A case study in a maritime pine forest in southwestern France. *European Journal of Soil Science* **61**, 807–822 (2010).

47 Batjes, N.H. Total carbon and nitrogen in the soils of the world. *European Journal of Soil Science* **47**, 151–163 (1996).

48 Jobbagy, E.G. & Jackson, R.B. The Vertical Distribution of Soil Organic Carbon and Its Relation to Climate and Vegetation. *Ecological Applications* **10**, 423-436 (2000).

49 Nelson, D. W. & Sommers, L. E. Total Carbon, Organic Carbon, and Organic Matter. In Methods of Soil Analysis, Part 3, Chemical Methods (ed D.L. Sparks), 961-1010 (Soil Science Society of America and American Society of Agronomy, Madison, WI, USA, 1996).

50 Elser, J. J. *et al.* Global analysis of nitrogen and phosphorus limitation of primary producers in freshwater, marine and terrestrial ecosystems. *Ecol. Lett.* **10**, 1135-1142 (2007).

51 Gurevitch, J. & Hedges, L. V. Statistical issues in ecological meta-analyses. *Ecology* **80**, 1142-1149 (1999).

52 Yanai, R. D., Arthur, M. A., Siccama, T. G. & Federer, C. A. Challenges of measuring forest floor organic matter dynamics: Repeated measures from a chronosequence. *For. Ecol. Manag.* **138**, 273-283 (2000).

(A)

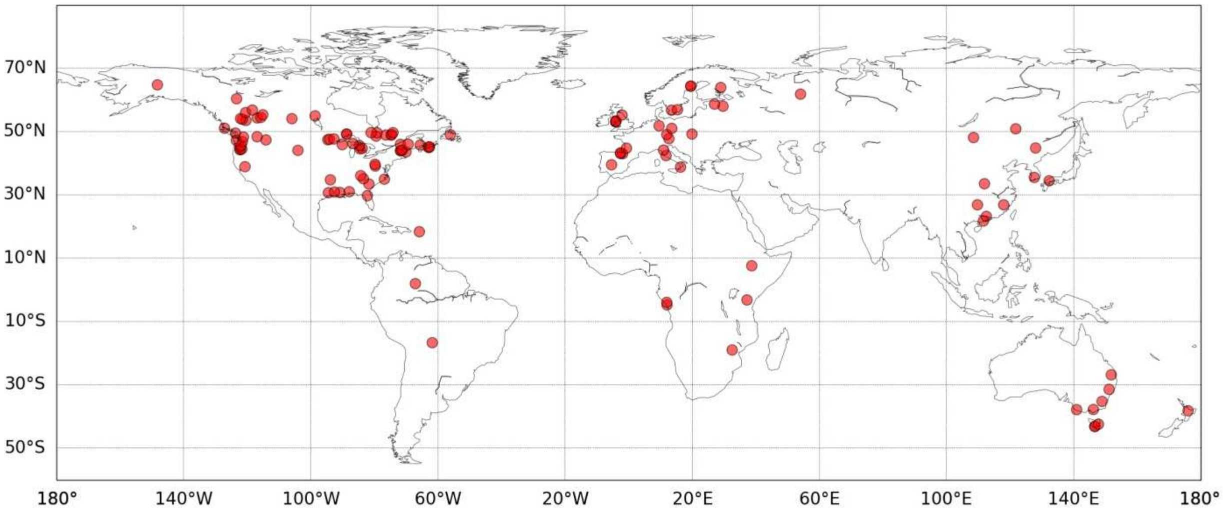

(B)

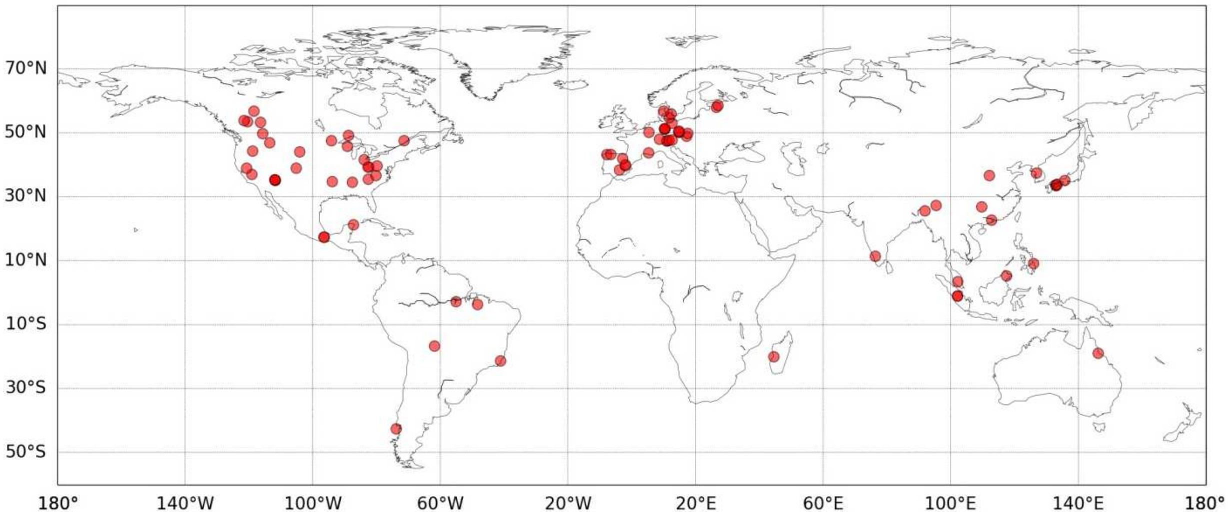

(C)

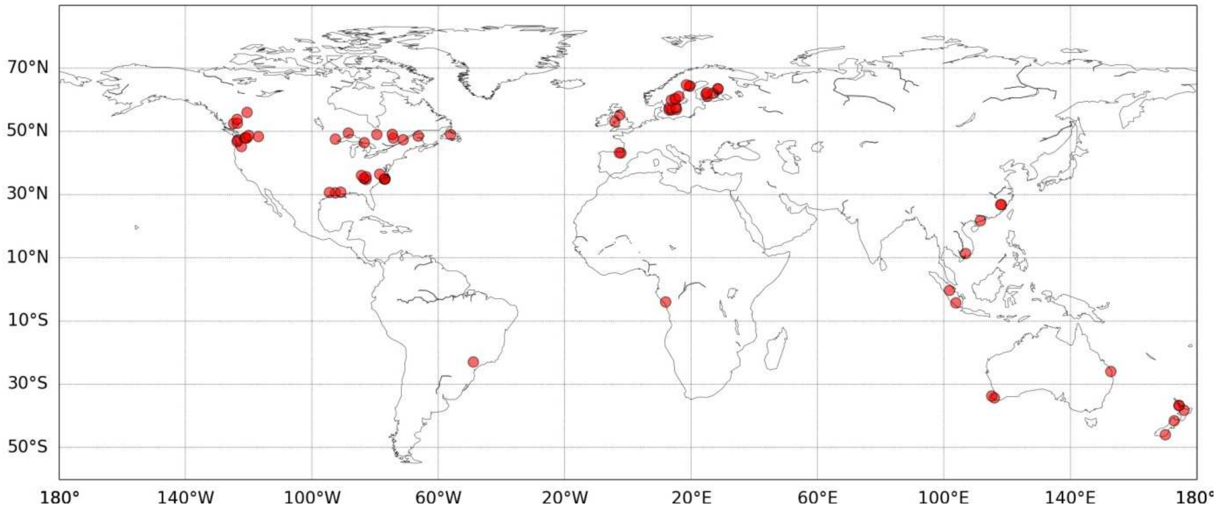

Fig. S1. Distribution of the study sites depending on harvest strategy. (A) Conventional clear-cutting (*N*=118 sites). (B) Conventional thinning (*N*=80 sites). (C) Intensive harvest (*N*=86 sites). Map created in Python Language version 2.7 (Python Software Foundation; www.python.org), using the *basemap* package (https://pypi.python.org/pypi/basemap/1.0.7) of the *matplotlib* library (http://matplotlib.org).

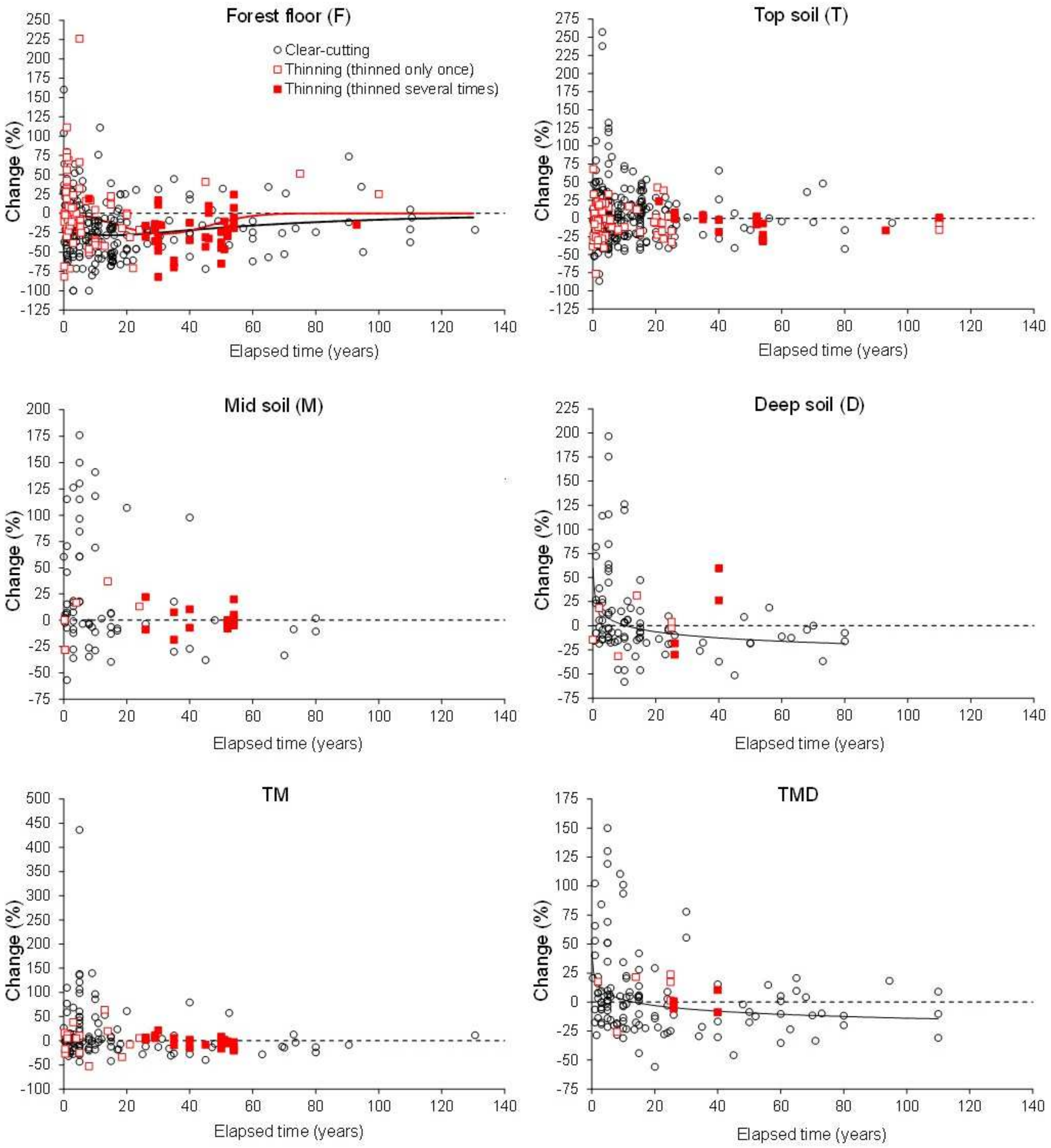

Fig. S2. Effects of conventional harvest on SOC stocks in different soil layers and as a function of the time elapsed since harvesting (complete dataset; 1,462 values). Values are expressed as percentage changes (comparison with unharvested controls). Contrary to the figures in the main text which present data with no pseudo-replicates, all data are shown here: a given site can be represented by several data points when different treatments were applied (e.g. clear-cutting with or without soil preparation following harvesting) or when SOC stocks were assessed at various times since harvesting. Relationships between the percentage change in forest floor (F) and time elapsed were fitted with a function adapted from Covington[20,52]:

$\%\ change = -12.63 \times time^{0.86} \times e^{-0.34 \times time^{0.56}}$ for clear-cutting (r^2=0.10, P<0.0001, n=214)

$\%\ change = -0.16 \times time^{1.64} \times e^{-2 \times 10^{-5} \times time^{2.96}}$ for thinning (r^2=0.11, P=0.003, n=110)

Based on the adjusted curves, SOC stocks in the forest floor decreased during the first 15-32 years (a decline of 26-28%) and then returned asymptotically to their initial values. There were also negative relationships between changes in SOC stocks in deep soil (D) and whole soil profile (TMD) and time elapsed (only for clear-cutting):

$\%\ change = \left(1.27 \times time^{-0.10} - 1\right) \times 100$ for deep soil (D, r^2=0.12, P=0.001, n=94)

$\%\ change = \left(1.21 \times time^{-0.07} - 1\right) \times 100$ for whole soil profile (TMD, r^2=0.09, P=0.001, n=122)

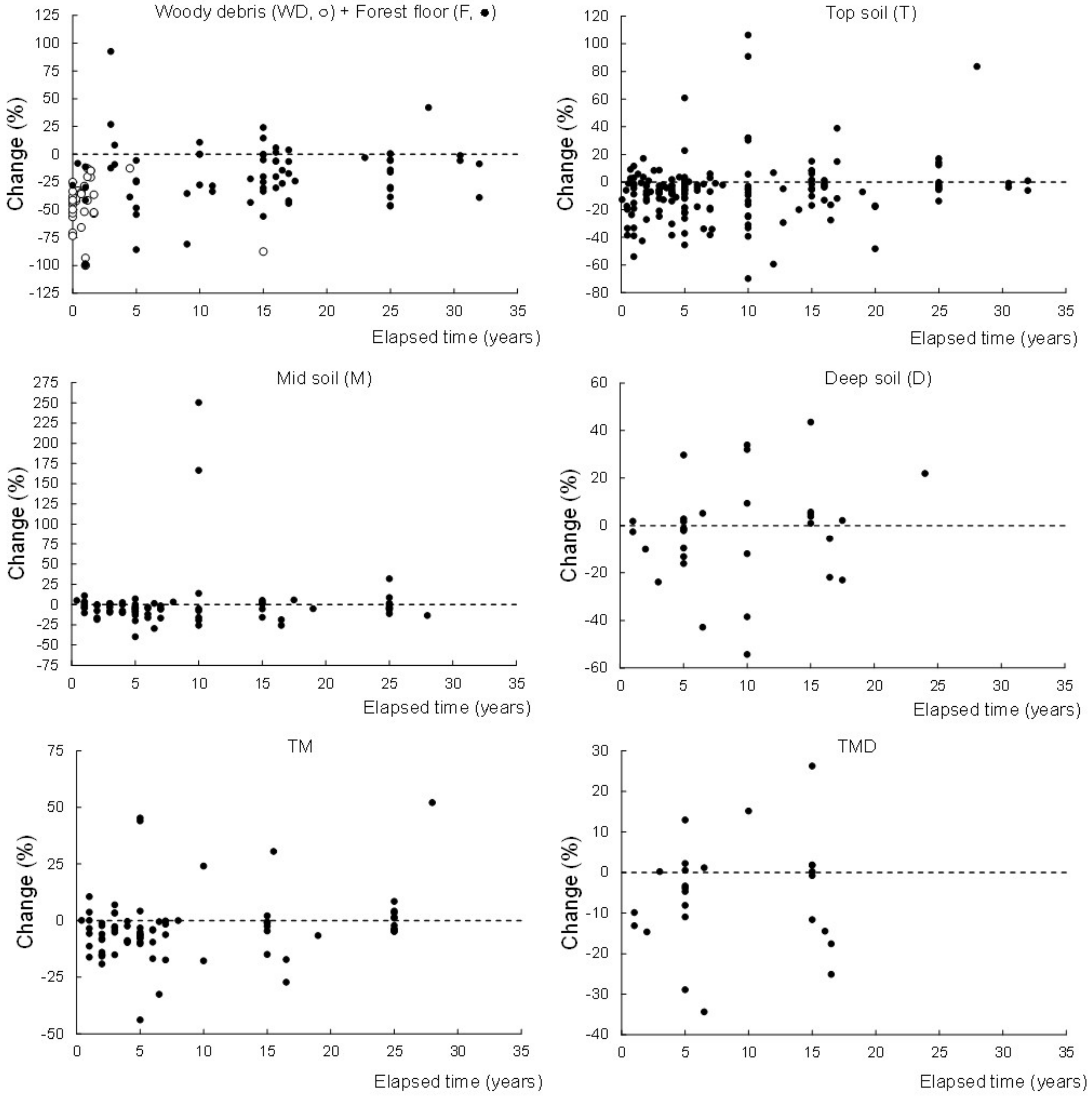

Fig. S3. Effects of intensive harvest on SOC stocks in different soil layers and as a function of the time elapsed since harvesting (complete dataset; 566 values). Values are expressed as percentage changes (comparison between whole-tree or whole-tree + forest floor harvest and stem-only harvest). Contrary to figures in the main text which present data with no pseudo-replicates, all data are shown here: a given site can be represented by several data points when different treatments were applied or when soil C stocks were assessed at various times since harvesting.

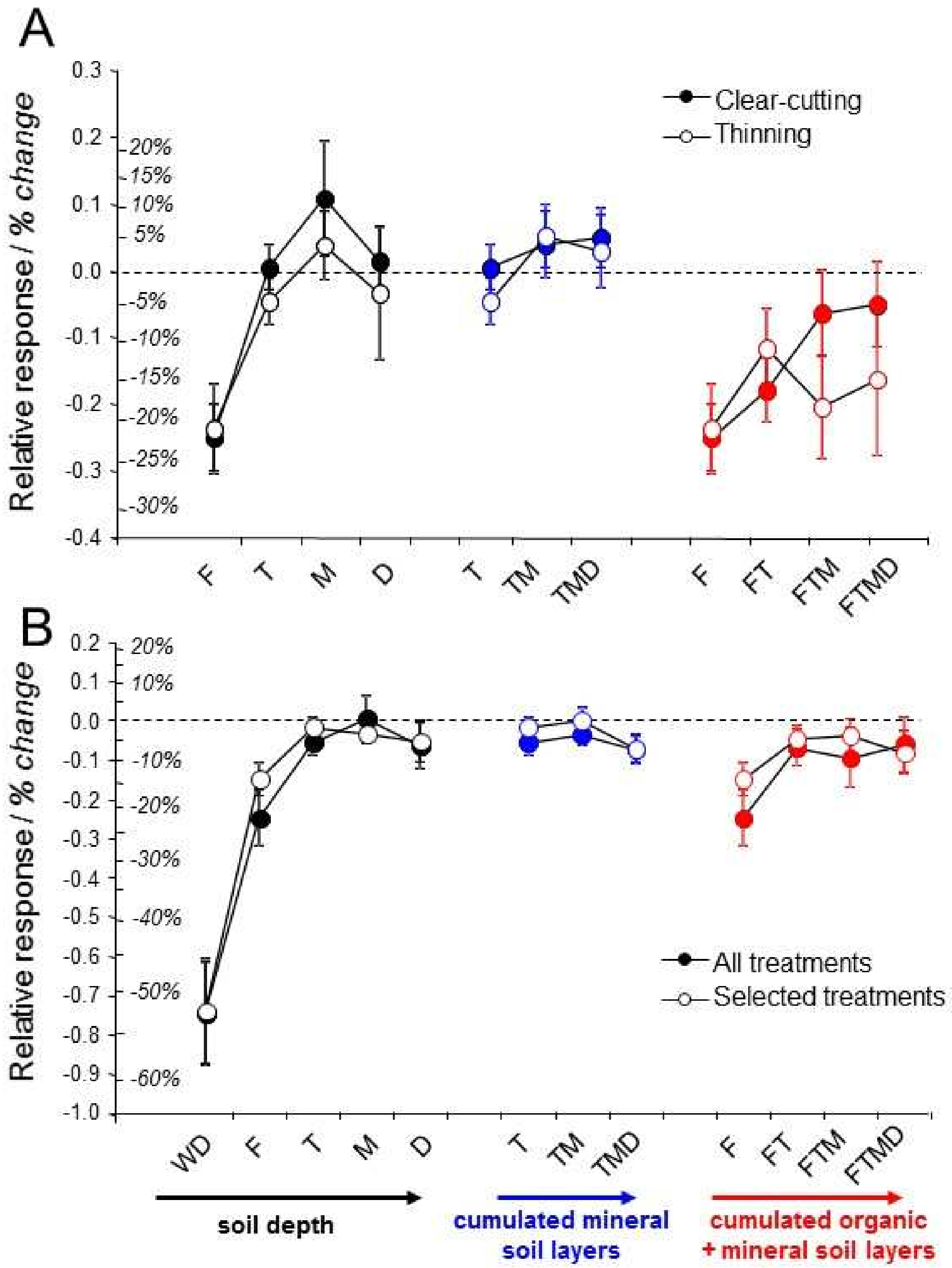

Fig. S4. Effects of conventional and intensive harvests on SOC stocks as a function of soil depth (individual soil layers) and thickness of soil profile (cumulated mineral soil layers or cumulated organic + mineral soil layers). (A) Conventional harvest at clear-cutting and thinning; **(B)** intensive harvest (whole-tree or whole-tree + forest floor harvests). For thinning, there was an additional effect of the thinning intensity (percentage of stems or basal area removed, see Fig. S5). There was however no effect of the number of thinning or management methods (selection cutting, shelterwood systems). Values (means ± standard errors) are expressed as relative responses: log(clear-cutting or thinning harvest/unharvested control) in (A), log(whole-tree or whole-tree + forest floor harvest/stem-only harvest) in (B). For the sake of clarity, comparisons between treatments and controls are also presented as the mean arithmetic difference (% higher or lower). Selected treatments in b correspond to whole-tree harvest (whole-tree + forest floor harvest were removed from the analysis). WD, woody debris; F, forest floor; T, top soil; M, mid soil; D, deep soil. Number of case studies (or sites) and *P* values are shown in Table S3.

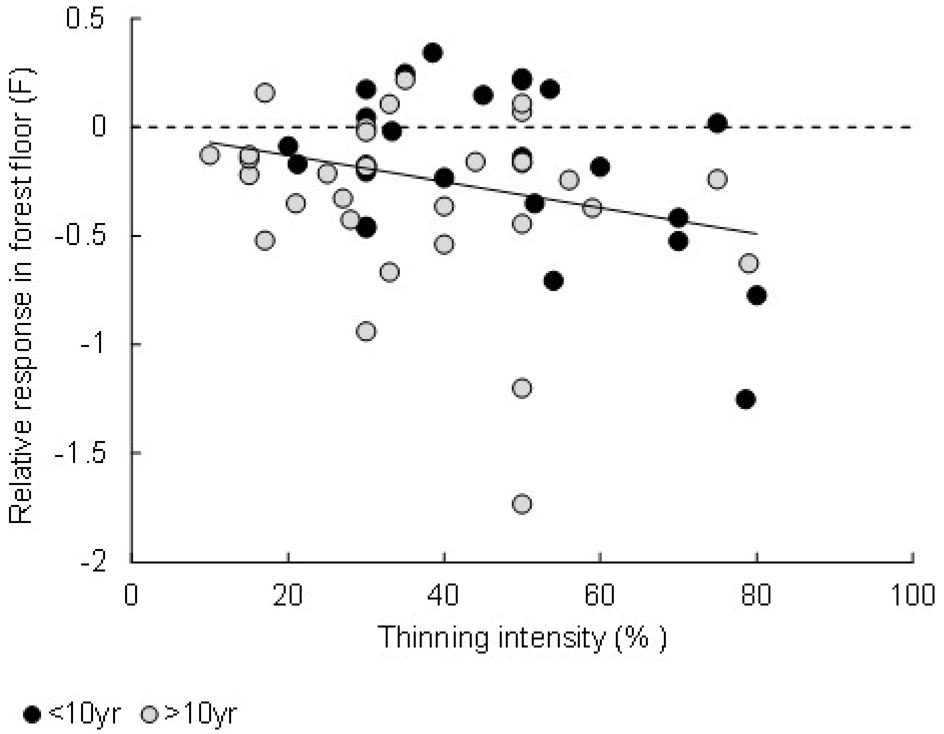

Fig. S5. Effects of conventional thinning on SOC stocks in the forest floor (F) relative to the thinning intensity. Values are expressed as relative responses [log(thinning harvest/unharvested control)]. The thinning intensity was the percentage of stand biomass removed (as estimated by stand basal area), or sometimes the percentage of trees removed, depending on the experimental design of case studies. The figure shows the linear regression for all data (linear regression for <10yr data: N=25, r^2=0.215, P=0.019; linear regression for >10yr data: N=32, P=0.197).

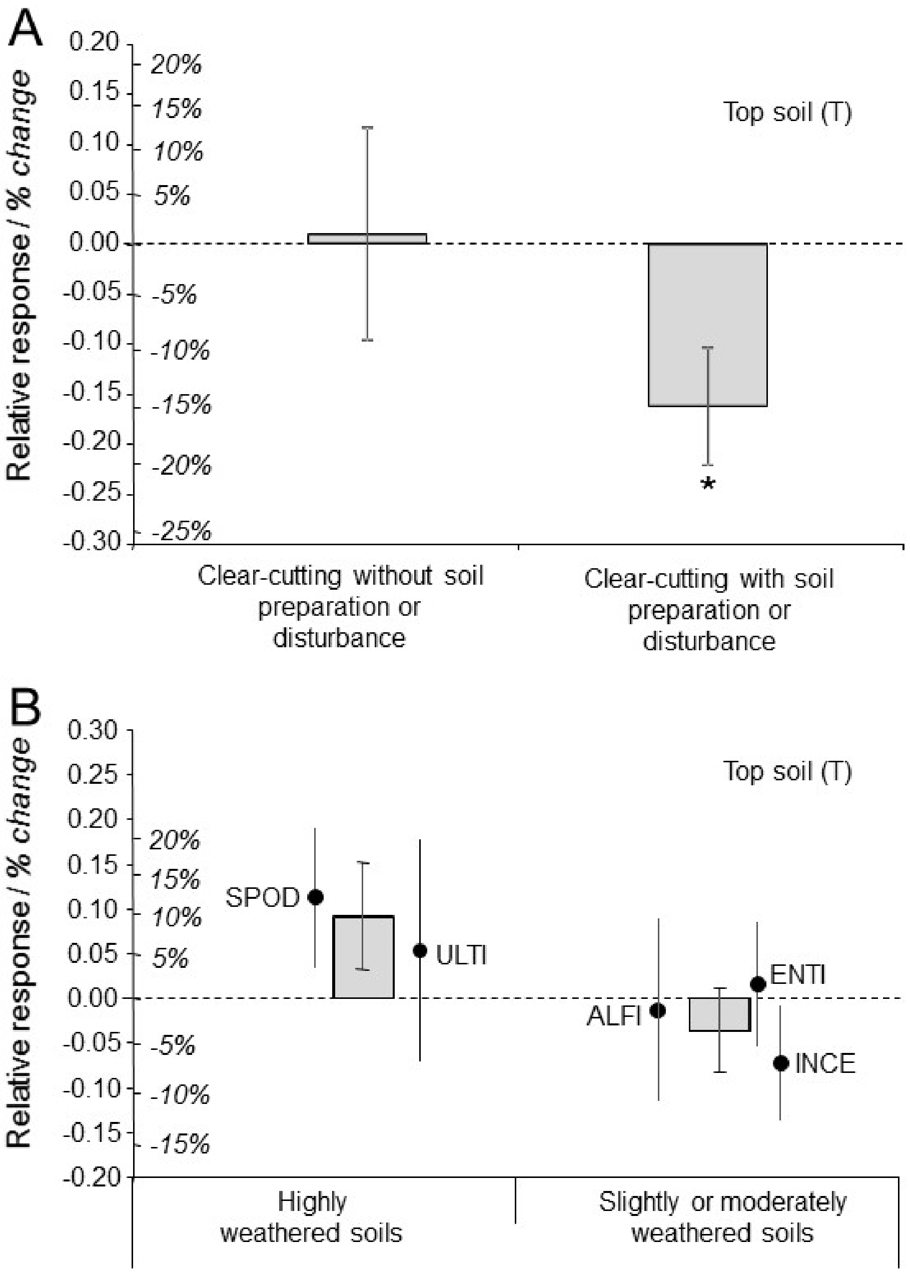

Fig. S6. Effects of conventional clear-cutting on SOC stocks in the top soil (T) layer (0-10 yrs after clear-cutting) depending on soil preparation (A) or soil types (B). Means ± standard errors. Values are expressed as relative response [log(clear-cutting harvest/unharvested control)]. For the sake of clarity, comparisons between treatments and controls are also presented as the mean arithmetic difference (% higher or lower). In (A), the number of case studies (or sites) is 17-28 and significant differences between relative responses and value 0 are denoted by an asterisk (*t* test). In (B), black symbols correspond to soil types (soil orders according to the USDA classification: SPOD, spodosols (N=16); ULTI, ultisols (N=7); ALFI, alfisols (N=9); ENTI, entisols (N=5); INCE, inceptisols (N=13) and grey bars correspond to soil classes (highly weathered soils (N=25, spodosols + ultisols + oxisols), slightly or moderately weathered soils (N=27, alfisols + entisols + inceptisols)). The relative response tends to be lower in slightly or moderately weathered soils than in highly weathered soils (comparison between soil classes with ANOVA: P=0.095). This was also the case for the effects of intensive harvest on C stocks in the forest floor (F) and top soil (T) (the relative response was lower in slightly and moderately weathered soils than in highly weathered soils: $P \leq 0.013$).

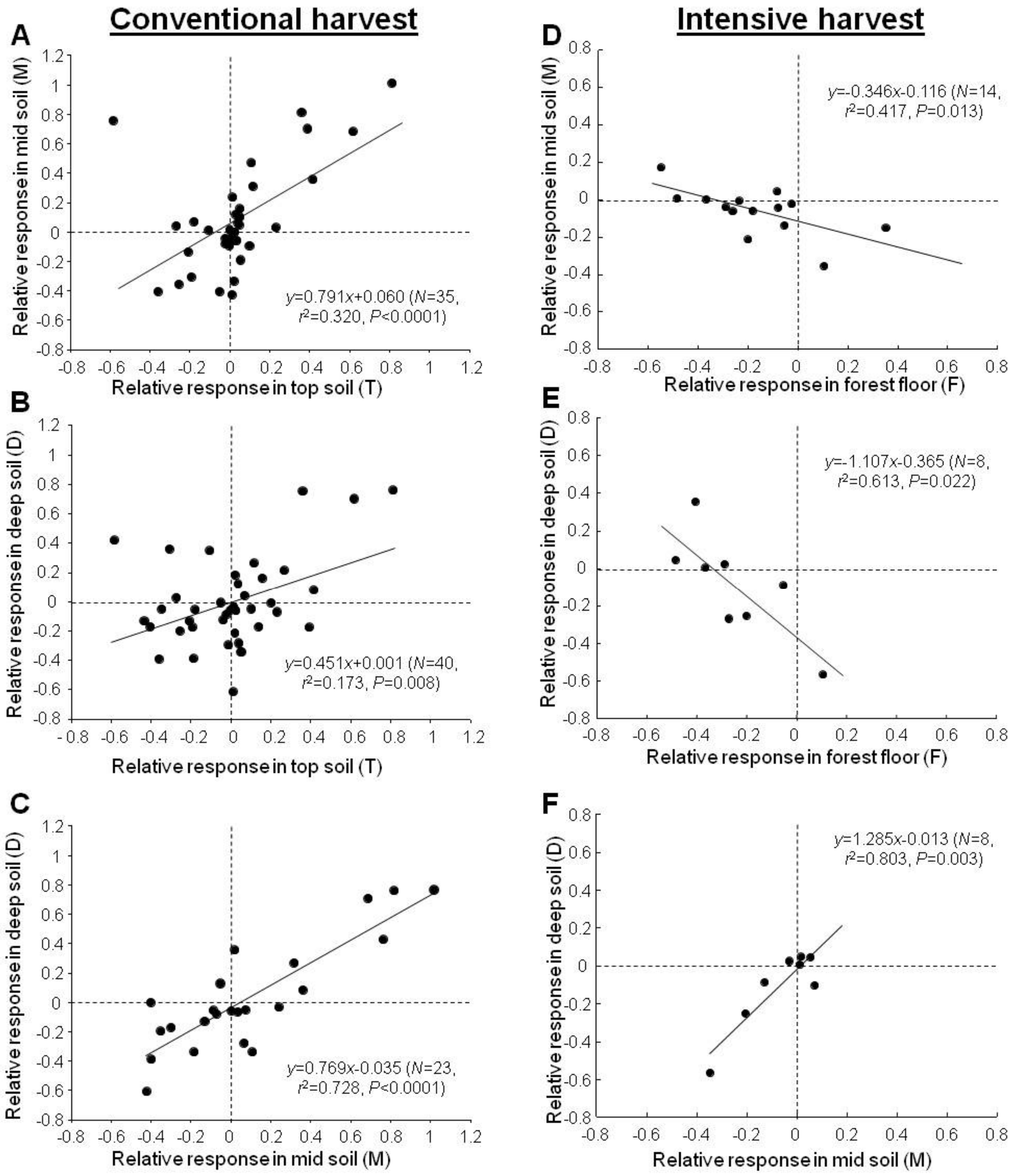

Fig. S7. Relationships among soil layers in the magnitude of change in SOC stocks in response to conventional (A-C) or intensive harvest (D-F). Values are expressed as relative responses: log(clear-cutting or thinning harvest/unharvested control) in A, B and C; log(whole-tree harvest/stem-only harvest) in D, E and F.

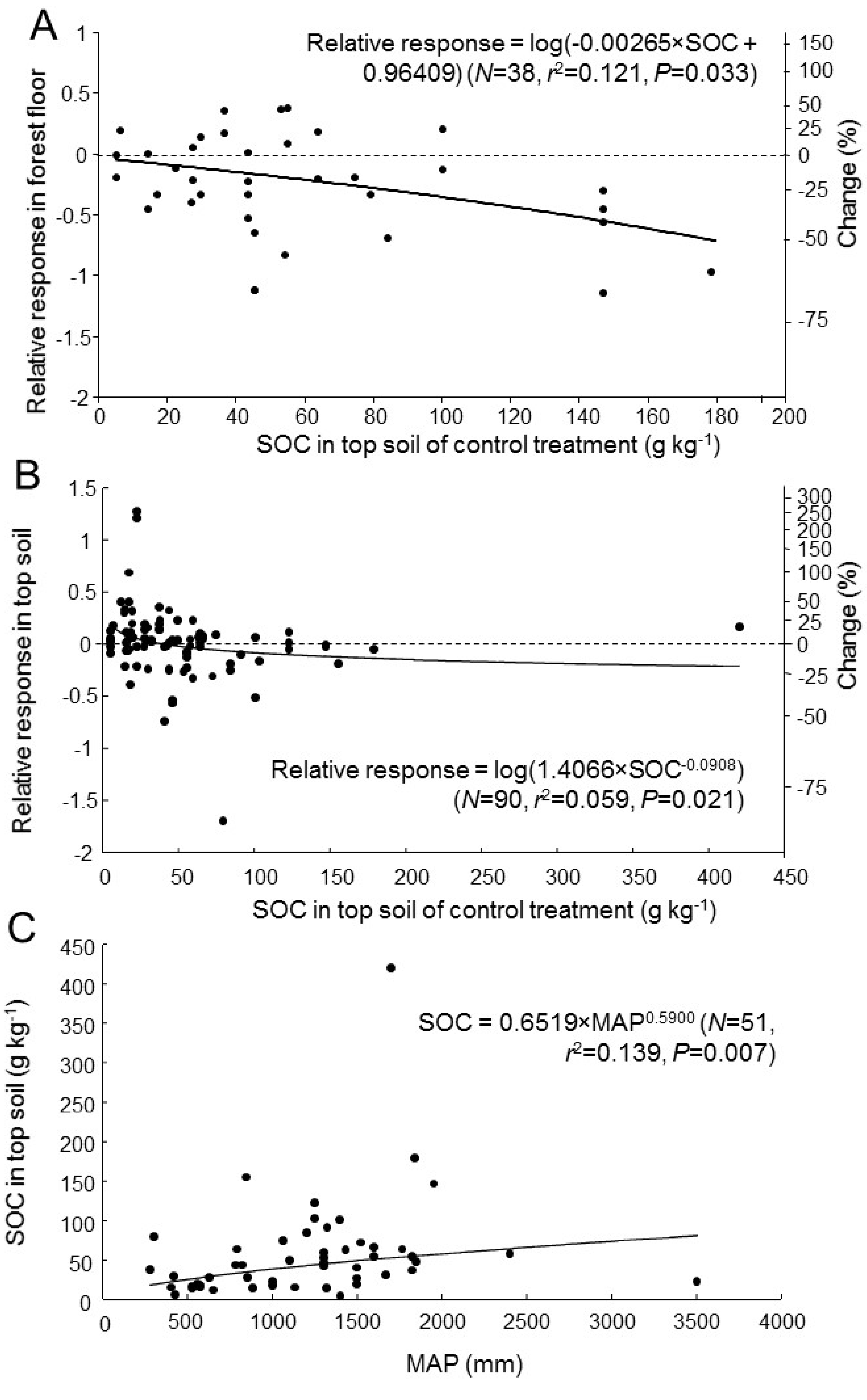

Fig. S8. Effects of conventional clear-cutting on SOC stocks in the forest floor (layer F; panel A) and top soil (layer T; panel B) relative to soil SOC concentration in the top soil of the control treatment. SOC concentration relative to the mean annual precipitation (MAP; panel C). Values are expressed as relative responses [log(clear-cutting harvest/unharvested control)]. For the sake of clarity, comparisons between treatments and controls are also presented as the mean arithmetic difference (% higher or lower) in panels A-B.

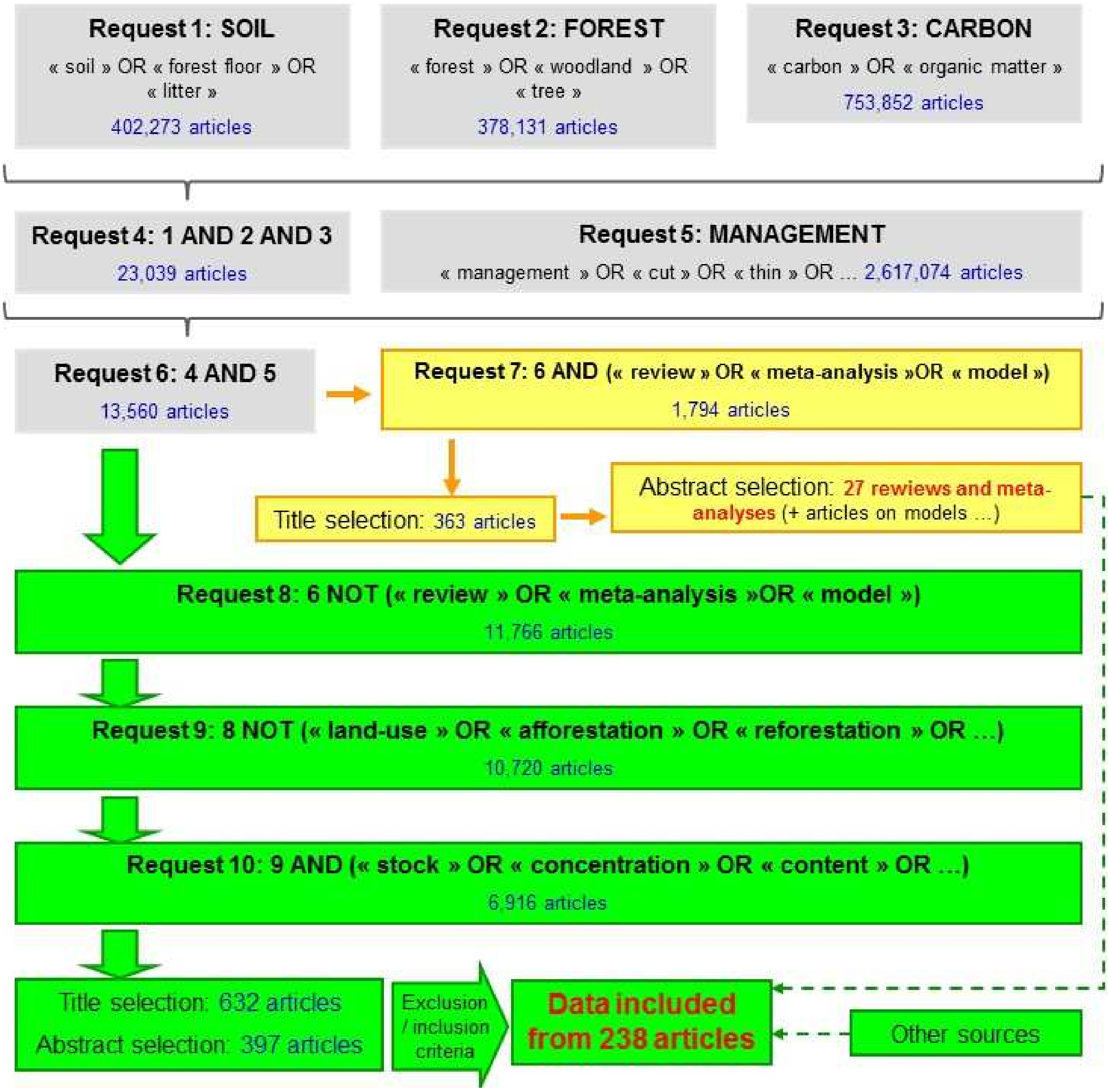

Fig. S9. Diagram showing the protocol used to select studies on the consequences of forest management on SOC stocks. Using the ISI Web of Science database and a series of bibliographic requests, we first identified a group of 13,560 records (grey boxes). Among these references, we identified reviews, meta-analyses and modelling studies in order to assess the current knowledge on SOC storage in forests (yellow boxes). In addition, we progressively sorted relevant case studies based on specific keywords, article title, and article abstract (397 publications). Finally, we used inclusion and exclusion criteria to select publications which reported relevant data (green boxes). A few items of data were obtained using other sources (mainly *Google scholar*) to find references cited in already existing reviews, meta-analyses or original publications. Examples of keywords used to select the publications are given between quotation marks and the number of records (number of publications) is given for each step.

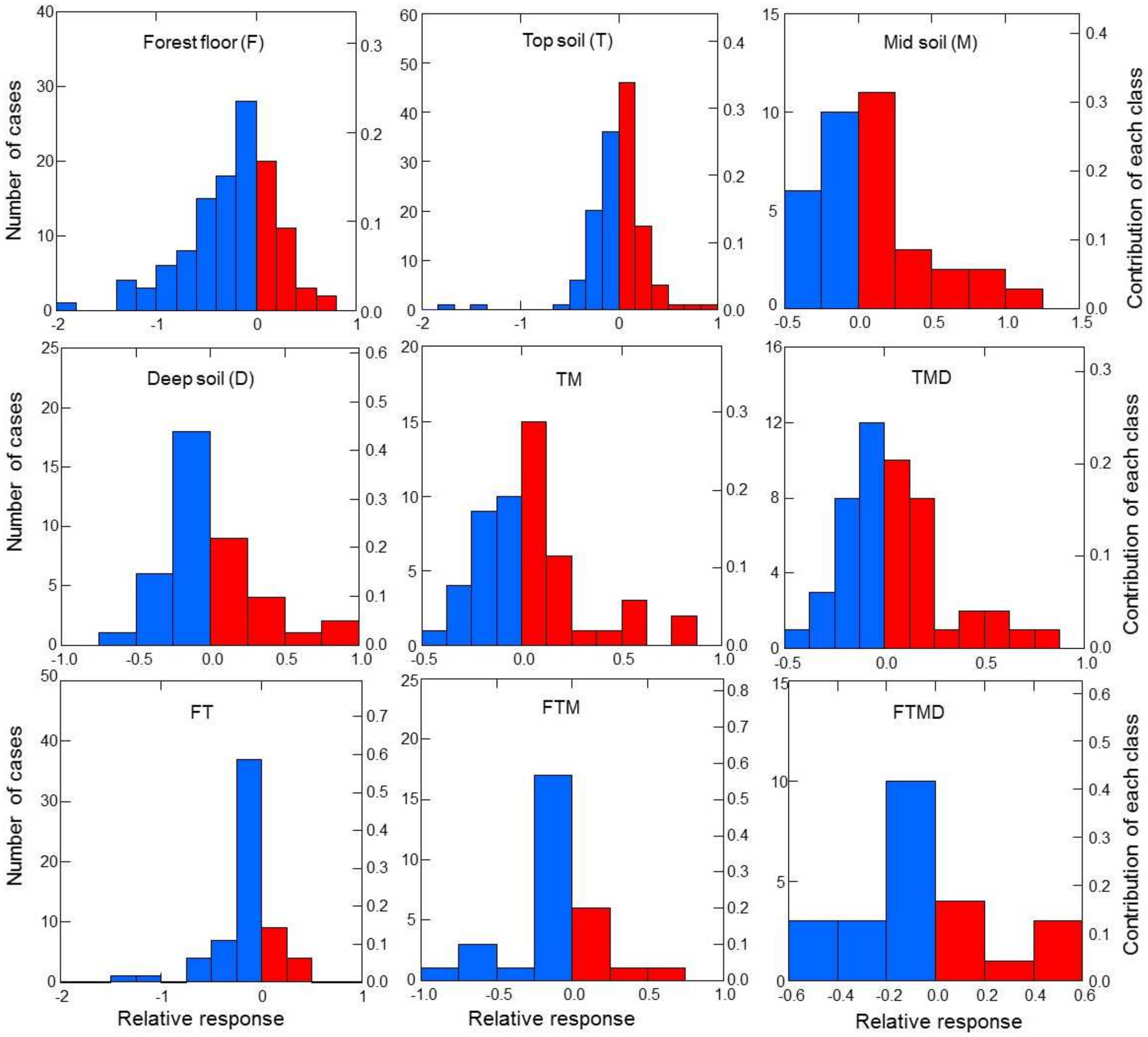

Fig. S10. Distribution of the case studies on the consequences of conventional harvest on SOC stocks relative to the value of the relative response. The relative response is the *log(clear-cutting or thinning harvest/unharvested control)* ratio. Blue and red colours indicate negative and positive responses, respectively.

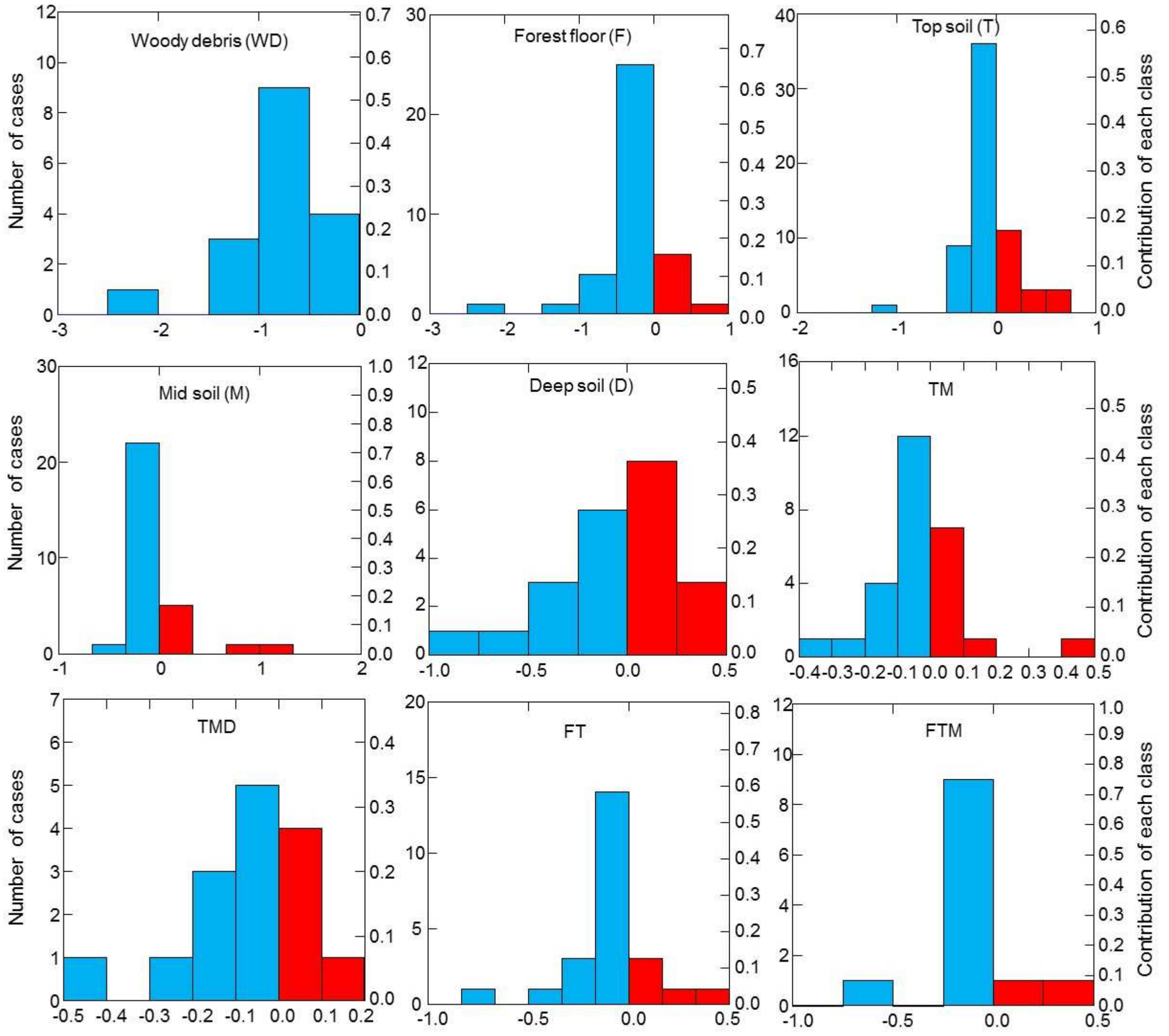

Fig. S11. Distribution of the case studies on the consequences of intensive harvest on SOC stocks relative to the value of the relative response. The relative response is the *log(whole-tree or whole-tree + forest floor harvest/stem-only harvest)* ratio. Blue and red colours indicate negative and positive responses, respectively.

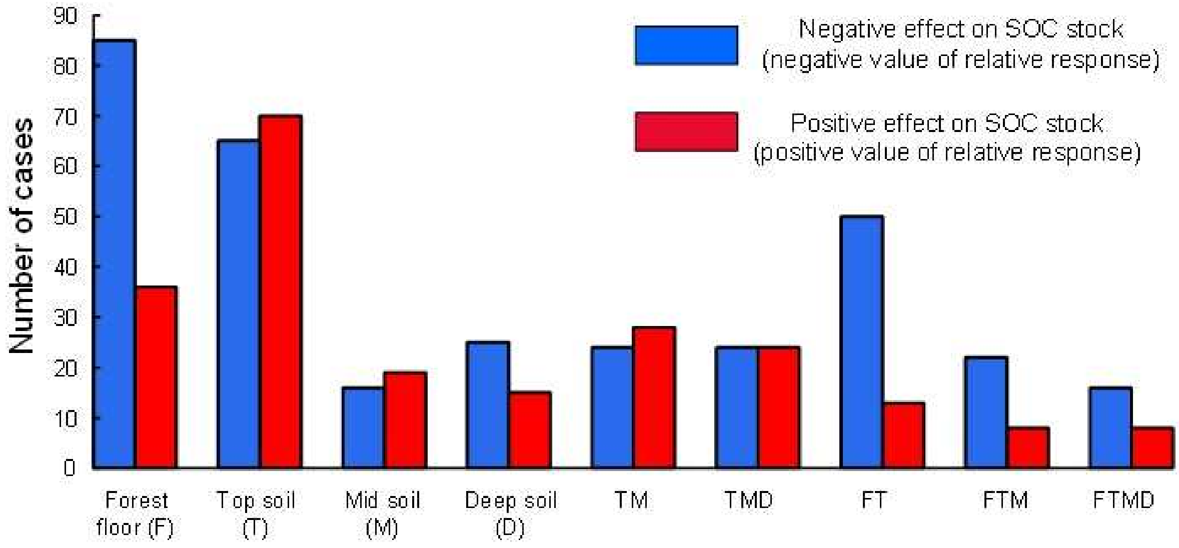

Fig. S12. Distribution of the case studies on the consequences of conventional harvest on SOC stocks relative to the value of the relative response (positive or negative).

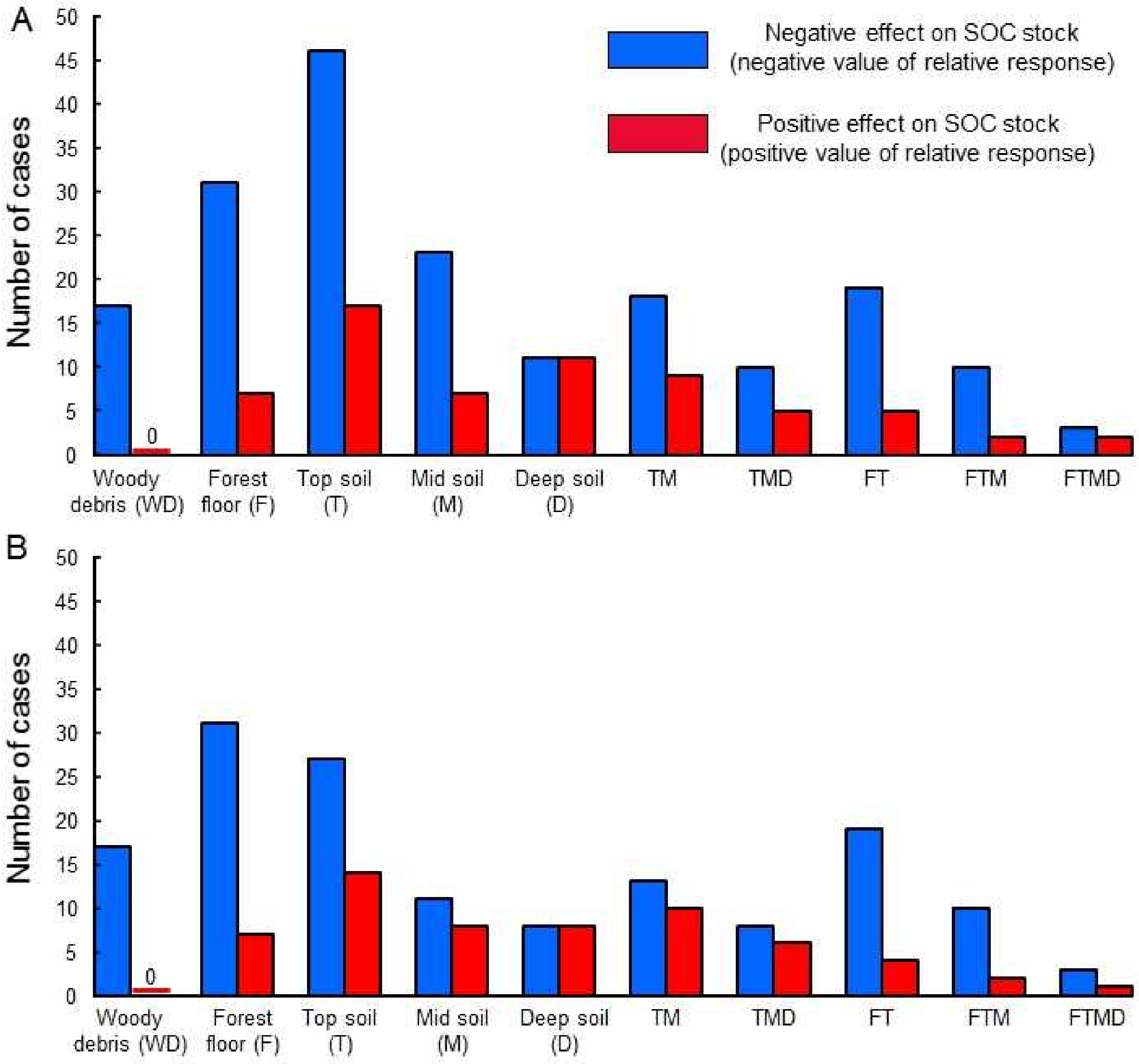

Fig. S13. Distribution of the case studies on the consequences of intensive harvest on SOC stocks relative to the value of the relative response (positive or negative). (**A**) All treatments; (**B**) selected treatments corresponding to whole-tree harvest (whole-tree + forest floor harvest removed from the analysis).

Table S1. Distribution of the study sites according to their geographical location, Koppen climate classes, harvest strategy, vegetation types and soil types. Number of case studies and contribution to total dataset (percentage in brackets).

	Conventional harvest		Intensive harvest
	Clear-cutting	Thinning	
Total dataset	**118 (100%)**	**80 (100%)**	**86 (100%)**
Location (Continent/State)			
North America			
Canada	30 (25%)	8 (10%)	14 (16%)
Mexico	0 (0%)	4 (5%)	0 (0%)
Puerto Rico	1 (1%)	0 (0%)	0 (0%)
USA	40 (34%)	20 (25%)	34 (40%)
**Total North America**	**71 (60%)**	**32 (40%)**	**48 (56%)**
South America			
Bolivia	1 (1%)	1 (1%)	0 (0%)
Brazil	0 (0%)	3 (4%)	1 (1%)
Chile	0 (0%)	1 (1%)	0 (0%)
Venezuela	1 (1%)	0 (0%)	0 (0%)
**Total South America**	**2 (2%)**	**5 (6%)**	**1 (1%)**
Europe			
Belgium	0 (0%)	1 (1%)	0 (0%)
Czech Republic	0 (0%)	5 (6%)	0 (0%)
Denmark	0 (0%)	3 (4%)	0 (0%)
Estonia	0 (0%)	1 (1%)	0 (0%)
Finland	1 (1%)	0 (0%)	7 (8%)
France	1 (1%)	1 (1%)	0 (0%)
Germany	4 (3%)	7 (9%)	0 (0%)
Italy	3 (3%)	0 (0%)	0 (0%)
Russia	1 (1%)	0 (0%)	0 (0%)
Slovakia	1 (1%)	0 (0%)	0 (0%)
Spain	4 (3%)	7 (9%)	1 (1%)
Sweden	4 (3%)	0 (0%)	12 (14%)
United Kingdom	4 (3%)	0 (0%)	2 (2%)
**Total Europe**	**23 (19%)**	**25 (31%)**	**22 (26%)**
Asia			
China	7 (6%)	3 (4%)	2 (2%)
India	0 (0%)	3 (4%)	0 (0%)
Indonesia	0 (0%)	1 (1%)	2 (2%)
Japan	1 (1%)	5 (6%)	0 (0%)
Korea	1 (1%)	1 (1%)	0 (0%)
Malaysia	0 (0%)	2 (3%)	0 (0%)
Mongolia	1 (1%)	0 (0%)	0 (0%)
Philippines	0 (0%)	1 (1%)	0 (0%)
Vietnam	0 (0%)	0 (0%)	1 (1%)
**Total Asia**	**10 (8%)**	**16 (20%)**	**5 (6%)**
Africa			
Congo	1 (1%)	0 (0%)	1 (1%)
Ethiopia	1 (1%)	0 (0%)	0 (0%)
Madagascar	0 (0%)	1 (1%)	0 (0%)
Tanzania	1 (1%)	0 (0%)	0 (0%)
Zimbabwe	1 (1%)	0 (0%)	0 (0%)
**Total Africa**	**4 (3%)**	**1 (1%)**	**1 (1%)**

Table S1 (Continued)

	Conventional harvest		Intensive harvest
	Clear-cutting	Thinning	
Location (Continent/State) (Continued)			
Oceania			
Australia	5 (4%)	1 (1%)	3 (3%)
New Zealand	1 (1%)	0 (0%)	6 (7%)
Tasmania	2 (2%)	0 (0%)	0 (0%)
**Total Oceania**	**8 (7%)**	**1 (1%)**	**9 (10%)**
Location (Hemisphere)			
Northern	106 (90%)	72 (90%)	73 (85%)
Southern	12 (10%)	8 (10%)	13 (15%)
Climate classes (Koeppen classification)			
Tropical	4 (3%)	12 (15%)	4 (5%)
Temperate	56 (47%)	46 (58%)	30 (35%)
Cold	58 (49%)	21 (26%)	35 (41%)
Polar	0 (0%)	1 (1%)	0 (0%)
Not specified	0 (0%)	0 (0%)	17 (20%)
Harvest strategy			
Clearcutting	118 (100%)	0 (0%)	79 (91%)
Thinning	0 (0%)	80 (100%)	8 (9%)
Pre-harvest vegetation			
Broad-leaved forests	41 (33%)	25 (31%)	16 (18%)
Coniferous forests	67 (54%)	47 (58%)	56 (64%)
Mixed forests	16 (13%)	9 (11%)	1 (1%)
Not specified	0 (0%)	0 (0%)	14 (16%)
Post-harvest vegetation			
Broad-leaved forests	35 (29%)	25 (31%)	16 (19%)
Coniferous forests	68 (56%)	47 (58%)	56 (65%)
Mixed forests	19 (16%)	9 (11%)	0 (0%)
Not specified	0 (0%)	0 (0%)	14 (16%)
Soil type (soil order according to the USDA classification)			
Histosols	1 (1%)	0 (0%)	0 (0%)
Spodosols	30 (25%)	2 (2%)	28 (33%)
Andisols	3 (2%)	2 (2%)	3 (3%)
Oxisols	1 (1%)	4 (5%)	2 (2%)
Ultisols	15 (12%)	13 (16%)	12 (14%)
Mollisols	0 (0%)	1 (1%)	0 (0%)
Alfisols	15 (12%)	8 (10%)	6 (7%)
Inceptisols	19 (16%)	16 (20%)	8 (9%)
Entisols	6 (5%)	10 (12%)	4 (5%)
Several soil types	16 (13%)	11 (14%)	4 (5%)
Not specified	15 (12%)	14 (17%)	19 (22%)

Table S2. Climate and characteristics of the study sites.

	Conventional harvest		Intensive harvest
	Clear-cutting	Thinning	
Latitude (°)			
Minimum	-43.2	-42.6	-46.0
Maximum	64.7	58.3	64.7
Mean	37.4	34.1	34.8
Median	45.4	39.5	47.9
Quartile Q1	35.0	31.9	30.6
Quartile Q3	49.4	47.8	56.9
Mean annual temperature (°C)			
Minimum	-3.4	-0.6	0.0
Maximum	26.0	29.5	28.4
Mean	8.6	11.7	8.9
Median	7.6	9.6	6.1
Quartile Q1	3.5	6.6	3.4
Quartile Q3	12.8	15.8	14.2
Mean annual precipitation (mm year^{-1})			
Minimum	280	320	440
Maximum	3500	3800	2800
Mean	1178	1279	1189
Median	1124	1070	1096
Quartile Q1	798	659	691
Quartile Q3	1412	1740	1478
Evapotranspiration (mm year^{-1})			
Minimum	272	227	309
Maximum	1461	1538	1557
Mean	609	689	649
Median	539	550	504
Quartile Q1	449	447	408
Quartile Q3	702	793	875
Altitude (m a.s.l.)			
Minimum	7	10	10
Maximum	2200	2850	1830
Mean	509	797	368
Median	390	575	248
Quartile Q1	224	192	135
Quartile Q3	711	1144	449
Age of pre-harvest vegetation (years)			
Minimum	4	7	6
Maximum	500	475	165
Mean	112	80	54
Median	83	50	45
Quartile Q1	50	21	27
Quartile Q3	126	99	78

Table S3. General effects of conventional and intensive harvests on C stocks as a function of soil depth (individual soil layers) or thickness of soil profile (cumulated soil layers). Number of case studies (or sites) and *P* values are shown.

Effects of conventional harvest

	Clear-cutting		Thinning	
	N_{sites}	P[¶]	N_{sites}	P[¶]
Forest floor (F)	67	**<0.0001**	52	**0.001**
Top soil (T)	85	0.879	51	0.186
Mid soil (M)	24	0.223	11	0.469
Deep soil (D)	33	0.800	8	0.744
TM	33	0.439	19	0.293
TMD	41	0.270	8	0.609
FT	37	**0.001**	26	**0.066**
FTM	18	0.335	12	**0.023**
FTMD	21	0.445	3	0.292

Effects of intensive harvest

	All treatments		Selected treatments[&]	
	N_{sites}	P[¶]	N_{sites}	P[¶]
Woody debris (WD)	17	**<0.0001**	17	**<0.0001**
Forest floor (F)	38	**0.001**	38	**0.001**
Top soil (T)	63	**0.081**	41	0.423
Mid soil (M)	30	0.981	20	0.152
Deep soil (D)	22	0.267	16	0.298
TM	27	0.153	23	0.924
TMD	15	**0.036**	14	**0.067**
FT	24	0.131	23	0.141
FTM	12	0.216	12	0.350
FTMD	5	0.398	4	0.211

[&]Selected treatments correspond to whole-tree harvest treatments (whole-tree + forest floor harvest removed from the analysis).

[¶]Differences between relative responses and value 0 were assessed using a *t* test.

References used in the meta-analysis

Effects of clear-cutting (conventional harvest) on soil C stocks

Abou Jaoudé, R., Lagomarsino, A. & De Angelis, P. Impacts of nitrogen fertilisation and coppicing on total and heterotrophic soil CO2 efflux in a short rotation poplar plantation. *Plant Soil* 339, 219–230 (2011).

Antisari, L.V., Falsone, G., Carbone, S. & Vianello, G. Short-term effects of forest recovery on soil carbon and nutrient availability in an experimental chestnut stand. *Biol. Fertil. Soils* 49, 165–173 (2013).

Archer, J.K., Miller, D.L. & Tanner, G.W. Changes in Understory Vegetation and Soil Characteristics following Silvicultural Activitiesin a Southeastern Mixed Pine Forest. *Journal of the Torrey Botanical Society* 134, 489–504 (2007).

Bauhus, J., Vor, T., Bartsch, N. & Cowling, A. The effects of gaps and liming on forest floor decomposition and soil C and N dynamics in a *Fagus sylvatica* forest. *Can. J. For. Res.* 34, 509–518 (2004).

Bélanger, N., Paré, D. & Yamasaki, S.H. The soil acid-base status of boreal black spruce stands after whole-tree and stem-only harvesting. *Can. J. For. Res.* 33, 1874–1879 (2003).

Bock, M.D. & Van Rees, K.C.J. Forest harvesting impacts on soil properties and vegetation communities in the Northwest Territories. *Can. J. For. Res.* 32, 713–724 (2002).

Bouillet, J.P., Nzila, J.D., Laclau, J.P. & Ranger, J. Effects of Site Management on Eucalyptus Plantations in the Equatorial Zone, on the Coastal Plains of the Congo. In: Nambiar, E.K.S., Tiarks, A., Cossalter, C. & Ranger, J. (Eds). Site Management and Productivity in Tropical Plantation Forests: A Progress Report, Workshop proceedings, Kerala, India, Published by Center for International Forestry Research Bogor, Indonesia, pp. 10–20 (2000).

Bradford, J.B. & Kastendick, D.N. Age-related patterns of forest complexity and carbon storage in pine and aspen-birch ecosystems of northern Minnesota, USA. *Can. J. For. Res.* 40, 401–409 (2010).

Brais, S., Paré, D., Camiré, C., Rochon, P. & Vasseur, C. Nitrogen net mineralization and dynamics following whole-tree harvesting and winter windrowing on clayey sites of northwestern Quebec. *For. Ecol. Manag.* 157, 119–130 (2002).

Brandtberg, P.O. & Olsson, B.A. Changes in the effects of whole-tree harvesting on soil chemistry during 10 years of stand development. *For. Ecol. Manag.* 277, 150–162 (2012).

Butnor, J.R., Johnsen, K.H. & Sanchez, F.G. Whole-tree and forest floor removal from a loblolly pine plantation have no effect on forest floor CO_2 efflux 10 years after harvest. *For. Ecol. Manag.* 227, 89–95 (2006).

Carter, M.C., Dean, T.J., Zhou, M., Messina, M.G. & Wang, Z. Short-term changes in soil C, N, and biota following harvesting and regeneration of loblolly pine (*Pinus taeda* L.). *For. Ecol. Manag.* 164, 67–88 (2002).

Chatterjee, A., Vance, G.F., Pendall, E. & Stahl, P.D. Timber harvesting alters soil carbon mineralization and microbial community structure in coniferous forests. *Soil Biol. Biochem.* 40, 1901–1907 (2008).

Chen, C.R., Xu, Z.H. & Mathers, N.J. Soil Carbon Pools in Adjacent Natural and Plantation Forests of Subtropical Australia. *Soil Sci. Soc. Am. J.* 68, 282–291 (2004).

Chen, H.Y.H. & Shrestha, B.M. Stand age, fire and clearcutting affect soil organic carbon and aggregation of mineral soils in boreal forests. *Soil Biol. Biochem.* 50, 149–157 (2012).

Christophel, D., Höllerl, S., Prietzel, J. & Steffens, M. Long-term development of soil organic carbon and nitrogen stocks after shelterwood- and clear-cutting in a mountain forest in the Bavarian Limestone Alps. *Eur. J. Forest Res.* 134, 623–640 (2015).

Clark, K.L., Gholz, H.L. & Castro, M.S. Carbon dynamics along a chronosequence of slash pine plantations in north Florida. *Ecol. Appl.* 14, 1154–1171 (2004).

Closa, I. & Goicoechea, N. Seasonal dynamics of the physicochemical and biological properties of soils in naturally regenerating, unmanaged and clear-cut beech stands in northern Spain. *Eur. J. Soil Biol.* 46, 190–199 (2010).

Covington, W.W. Changes in the forest floor organic matter and nutrient content following clear cutting in northern hardwoods. *Ecology* 62, 41–48 (1981).

Dai, K.O.H., Johnson, C.E. & Driscoll, C.T. Organic matter chemistry and dynamics in clear-cut and unmanaged hardwood forest ecosystems. *Biogeochemistry* 54, 51–83 (2001).

Das Gupta, S. & DeLuca, T.H. Short-term changes in belowground C, N stocks in recently clear felled Sitka spruce plantations on podzolic soils of North Wales. *For. Ecol. Manag.* 281, 48–58 (2012).

Diochon, A., Kellman, L. & Beltrami, H. Looking deeper: An investigation of soil carbon losses following harvesting from a managed northeastern red spruce (*Picea rubens Sarg.)* forest chronosequence. *For. Ecol. Manag.* 257, 413–420 (2009).

Don, A. *et al.* No rapid soil carbon loss after a windthrow event in the High Tatra. *For. Ecol. Manag.* 276, 239–246 (2012).

Edeso, J.M., Merino, A., Gonzalez, M.J. & Marauri, P. Soil erosion under different harvesting managements in steep forestlands from northern Spain. *Land Degrad. Develop.* 10, 79–88 (1999).

Edmonds, R.L. & McColl, J.G. Effects of Forest Management on Soil Nitrogen in *Pinus radiata* Stands in the Australian Capital Territory. *For. Ecol. Manag.* 29, 199–212 (1989).

Ellis, R.C. & Graley, A.M. Gains and losses in soil nutrients associated with harvesting and burning eucalypt rainforest. *Plant Soil* 74, 437–450 (1983).

Ellis, R.C., Lowry, R.K. & Davies, S.K. The effect of regeneration burning upon the nutrient status of soil in two forest types in southern Tasmania. *Plant Soil* 65,171–186 (1982).

Epron, D., Nouvellon, Y., Deleporte, P. *et al.* Soil carbon balance in a clonal Eucalyptus plantation in Congo: effects of logging on carbon inputs and soil CO_2 efflux. *Glob. Change Biol.* 12, 1021–1031 (2006).

Falsone, G., Celi, L., Caimi, A., Simonov, G. & Bonifacio, E. The effect of clear cutting on podzolisation and soil carbon dynamics in boreal forests (Middle Taiga zone, Russia). *Geoderma* 177–178, 27–38 (2012).

Federer, C.A. Organic matter and nitrogen content of the forest floor in even-aged northern hardwoods. *Can. J. For. Res.* 14, 763–767 (1984).

Fredeen, A.L., Bois, C.H., Janzen, D.T. & Sanborn, P.T. Comparison of coniferous forest carbon stocks between old-growth and young second-growth forests on two soil types in central British Columbia, Canada. *Can. J. For. Res.* 35: 1411–1421 (2005).

Gartzia-Bengoetxea, N., Gonzalez-Arias, A. & Martinez de Arano, I. Effects of tree species and clear-cut forestry on forest floor characteristics in adjacent temperate forests in Northern Spain. *Can. J. For. Res.* 39, 1302–1312 (2009).

Gartzia-Bengoetxea, N., Gonzalez-Arias, A., Merino, A. & Martinez de Arano, I. Soil organic matter in soil physical fractions in adjacent semi-natural and cultivated stands in temperate Atlantic forests. *Soil Biol. Biochem.* 41, 1674–1683 (2009).

Gilliam, F.S. Effects of harvesting on herbaceous layer diversity of a central Appalachian hardwood forest in West Virginia, USA. *For. Ecol. Manag.* 155, 33–43 (2002).

Gough, C.M., Vogel, C.S., Harrold, K.H., George, K. & Curtis, P.S. The legacy of harvest and fire on ecosystem carbon storage in a north temperate forest. *Glob. Change Biol.* 13, 1935–1949 (2007).

Grand, S. & Lavkulich, L.M. Effects of Forest Harvest on Soil Carbon and Related Variables in Canadian Spodosols. *Soil Sci. Soc. Am. J.* 76, 1816–1827 (2012).

Griffiths, R.P., Gray, A.N. & Spies, T.A. Soil Properties in Old-growth Douglas-fir Forest Gaps in the Western Cascade Mountains of Oregon. *Northwest Sci.* 84, 33–45 (2010).

Griffiths, R.P. & Swanson, A.K. Forest soil characteristics in a chronosequence of harvested Douglas-fir forests. *Can. J. For. Res.* 31, 1871–1879 (2001).

Hannam, K.D., Quideau, S.A., Kishchuk, B.E., Oh, S.W. & Wasylishen, R.E. Forest-floor chemical properties are altered by clear-cutting in boreal mixedwood forest stands dominated by trembling aspen and white spruce. *Can. J. For. Res.* 35: 2457–2468 (2005).

Hazlett, P.W., Gordon, A.M., Voroney, R.P. & Sibley, P.K. Impact of harvesting and logging slash on nitrogen and carbon dynamics in soils from upland spruce forests in northeastern Ontario. *Soil Biol. Biochem.* 39, 43–57 (2007).

Hoover, C.M., Leak, W.B. & Keel, B.G. Benchmark carbon stocks from old-growth forests in northern New England, USA. *For. Ecol. Manag.* 266, 108–114 (2012).

Huang, Z. *et al.* Harvest residue management effects on tree growth and ecosystem carbon in a Chinese fir plantation in subtropical China. *Plant Soil* 364, 303–314 (2013).

Johnson, C.E., Johnson, A.H., Huntington, T.G. & Siccama, T.G. Whole-Tree Clear-Cutting Effects on Soil Horizons and Organic-Matter Pools. *Soil Sci. Soc. Am. J.* 55, 497–502 (1991).

Johnson, C.E., Johnson, A.H. & Siccama, T.G. Whole-Tree Clear-Cutting Effects on Exchangeable Cations and Soil Acidity. *Soil Sci. Soc. Am. J.* 55, 502–508 (1991).

Johnson, D.W. & Todd, D.E. Harvesting Effects on Long-Term Changes in Nutrient Pools of Mixed Oak Forest. *Soil Sci. Soc. Am. J.* 62, 1725–1735 (1998).

Johnson, D.W., Todd, D.E. & Tolbert, V.R. Changes in Ecosystem Carbon and Nitrogen in a Loblolly Pine Plantation over the First 18 Years. *Soil Sci. Soc. Am. J.* 67, 1594–1601 (2003).

Jolivet, C. Le carbone organique des sols des Landes de Gascogne. Variabilité spatiale et effets des pratiques sylvicoles et agricoles. PhD-Thesis, Université de Bourgogne and INRA d'Orléans, pp. 313 (2000).

Jolivet, C., Angers, D.A., Chantigny, M.H., Andreux, F. & Arrouays, D. Carbohydrate dynamics in particle-size fractions of sandy spodosols following forest conversion to maize cropping. *Soil Biol. Biochem.* 38, 2834–2842 (2006).

Jones, H.S., Beets, P.N., Kimberley, M.O. & Garrett, L.G. Harvest residue management and fertilisation effects on soil carbon and nitrogen in a 15-year-old *Pinus radiata* plantation forest. *For. Ecol. Manag.* 262, 339–347 (2011).

Kabzems, R. & Haeussler, S. Soil properties, aspen, and white spruce responses 5 years after organic matter removal and compaction treatments. *Can. J. For. Res.* 35, 2045–2055 (2005).

Keenan, R.J., Messier, C. & Kimmins, J.P. Effects of clearcutting and soil mixing on soil properties and understorey biomass in western red cedar and western hemlock forests on northern Vancouver Island, Canada. *For. Ecol. Manag.* 68, 251–261 (1994).

Kennard, D.K. & Gholz, H.L. Effects of high- and low-intensity fires on soil properties and plant growth in a Bolivian dry forest. *Plant Soil* 234, 119–129 (2001).

Kim, C. Soil CO2 efflux in clear-cut and uncut red pine (*Pinus densiflora* S. et Z.) stands in Korea. *For. Ecol. Manag.* 255, 3318–3321 (2008).

Klockow, P.A., D'Amato, A.W. & Bradford, J.B. Impacts of post-harvest slash and live-tree retention on biomass and nutrient stocks in *Populus tremuloides* Michx.-dominated forests, northern Minnesota, USA. *For. Ecol. Manag.* 291, 278–288 (2013).

Klopatek, J.M. Belowground carbon pools and processes in different age stands of Douglas-fir. *Tree Physiol.* 22, 197–204 (2002).

Knoepp, J.D. & Swank, W.T. Forest Management Effects on Surface Soil Carbon and Nitrogen. *Soil Sci. Soc. Am. J.* 61, 928–935 (1997).

Krause, H. Forest floor mass and nutrients in two chronosequences of plantations: Jack pine vs. black spruce. *Can. J. Soil Sci.* 78, 77–83 (1998).

Laclau, J.P. *et al.* Organic residue mass at planting is an excellent predictor of tree growth in Eucalyptus plantations established on a sandy tropical soil. *For. Ecol. Manag.* 260, 2148–2159 (2010).

Laiho, R., Sanchez, F., Tiarks, A., Dougherty, P.M. & Trettin, C.C. Impacts of intensive forestry on early rotation trends in site carbon pools in the southeastern US. *For. Ecol. Manag.* 174, 177–189 (2003).

Law, B.E., Sun, O.J., Campbell, J., Vantuyl, S. & Thornton, P.E. Changes in carbon storage and fluxes in a chronosequence of ponderosa pine. *Glob. Change Biol.* 9, 510–524 (2003).

LeDuc, S.D. & Rothstein, D.E. Initial recovery of soil carbon and nitrogen pools and dynamics following disturbance in jack pine forests: A comparison of wildfire and clearcut harvesting. *Soil Biol. Biochem.* 39, 2865–2876 (2007).

Lee, J., Morrison, I.K., Leblanc, J.D., Dumas, M.T. & Cameron, D.A. Carbon sequestration in trees and regrowth vegetation as affected by clearcut and partial cut harvesting in a second-growth boreal mixedwood. *For. Ecol. Manag.* 169, 83–101 (2002).

Leggett, Z.H. & Kelting, D.L. Fertilization Effects on Carbon Pools in Loblolly Pine Plantations on Two Upland Sites. *Soil Sci. Soc. Am. J.* 70, 279–286 (2006).

Liechty, H.O., Shelton, M.G., Luckow, K.R. & Turton, D.J. Impacts of Shortleaf Pine-Hardwood Forest Management on Soils in the Ouachita Highlands: A Review. *South. J. Appl. For.* 26, 43–51 (2002).

Lindo, Z. & Visser, S. Microbial biomass, nitrogen and phosphorus mineralization, and mesofauna in boreal conifer and deciduous forest floors following partial and clear-cut harvesting. *Can. J. For. Res.* 33, 1610–1620 (2003).

Luan, J., Liu, S., Zhu, X. & Wang, J. Soil carbon stocks and fluxes in a warm-temperate oak chronosequence in China. *Plant Soil* 347, 243–253 (2011).

Ludwig, B., Khanna, P.K., Raison, R.J. & Jacobsen, K. Modelling changes in cation composition of a soil after clearfelling a eucalypt forest in East Gippsland, Australia. *Geoderma* 80, 95–116 (1997).

Mallik, A.U. & Hu, D. Soil respiration following site preparation treatments in boreal mixedwood forest. *For. Ecol. Manag.* 97, 265–275 (1997).

Mammen, A.v., Bachmann, M., Prietzel, J., Pretzsch, H. & Rehfuess, K.E. Bodenzustand, Ernährungszustand und Wachstum von Fichten (*Picea abies* Karst.) auf Probeflächen des Friedenfelser Verfahrens in der Oberpfalz. Soil Chemistry, Nutrition and Growth of Norway Spruce (*Picea abies* Karst.) on Sample Sites of the Friedenfels (Upper Palatia, Germany) Amelioration Experiment. *Forstw. Cbl.* 122, 99−114 (2003).

Mariani, L., Chang, S.X. & Kabzems, R. Effects of tree harvesting, forest floor removal, and compaction on soil microbial biomass, microbial respiration, and N availability in a boreal aspen forest in British Columbia. *Soil Biol. Biochem.* 38, 1734–1744 (2006).

Martin, J.L., Gower, S.T., Plaut, J. & Holmes, B. Carbon pools in a boreal mixedwood logging chronosequence. *Glob. Change Biol.* 11, 1883–1894 (2005).

Matsuzaki, E., Sanborn, P., Fredeen, A.L., Shaw, C.H. & Hawkins, C. Carbon stocks in managed and unmanaged old-growth western redcedar and western hemlock stands of Canada's inland temperate rainforests. *For. Ecol. Manag.* 297, 108–119 (2013).

Mattson, K.G. & Smith, H.C. Detrital organic matter and soil CO_2 efflux in forests regenerating from cutting in west Virginia. *Soil Biol. Biochem.* 25, 1241–1248 (1993).

Mattson, K.G. & Swank, W.T. Soil and detrital carbon dynamics following forest cutting in the Southern Appalachians. *Biol. Fertil. Soils* 7, 247–253 (1989).

May, B.M. & Attiwill, P.M. Nitrogen-fixation by *Acacia dealbata* and changes in soil properties 5 years after mechanical disturbance or slash-burning following timber harvest. *For. Ecol. Manag.* 181, 339–355 (2003).

McKee, S.E. *et al.* Carbon pools and fluxes in a tupelo (*Nyssa aquatica*)-baldcypress (*Taxodium distichum*) swamp 24-years after harvest disturbances. *Biomass Bioenerg.* 55, 130–140 (2013).

McLaughlin, J.W. & Phillips, S.A. Soil carbon, nitrogen, and base cation cycling 17 years after whole-tree harvesting in a low-elevation red spruce (*Picea rubens*)-balsam fir (*Abies balsamea*) forested watershed in central Maine, USA. *For. Ecol. Manag.* 222, 234–253 (2006).

Merino, A. & Edeso, J.M. Soil fertility rehabilitation in young *Pinus radiata* D. Don. Plantations from northern Spain after intensive site preparation. *For. Ecol. Manag.* 116, 83–91 (1999).

Merino, A., Edeso, J.M., Gonzalez, M.J. & Marauri, P. Soil properties in a hilly area following different harvesting management practices. *For. Ecol. Manag.* 103, 235–246 (1998).

Mujuru, L., Gotora, T., Velthorst, E.J., Nyamangara, J. & Hoosbeek, M.R. Soil carbon and nitrogen sequestration over an age sequence of *Pinus patula* plantations in Zimbabwean Eastern Highlands. *For. Ecol. Manag.* 313, 254–265 (2014).

Mummey, D.L. *et al.* Spatial analysis reveals differences in soil microbial community interactions between adjacent coniferous forest and clearcut ecosystems. *Soil Biol. Biochem.* 42, 1138–1147 (2010).

Muscolo, A., Sidari, M. & Mercurio, R. Influence of gap size on organic matter decomposition, microbial biomass and nutrient cycle in Calabrian pine (*Pinus laricio*, Poiret) stands. *For. Ecol. Manag.* 242, 412–418 (2007).

O'Brien, N.D., Attiwill, P.M. & Weston, C.J. Stability of soil organic matter in *Eucalyptus regnans* forests and *Pinus radiata* plantations in south eastern Australia. *For. Ecol. Manag.* 185, 249–261 (2003).

Olsson, B.A., Staaf, H., Lundkvist, H., Bengtsson, J. & Rosen, K. Carbon and nitrogen in coniferous forest soils after clear-felling and harvests of different intensity. *For. Ecol. Manag.* 82, 19–32 (1996).

Park, Y.D., Lee, D.K., Stanturf, J.A., Woo, S.Y. & Zoyo, D. Ecological Indicators of Forest Degradation after Forest Fire and Clear-cutting in the Siberian Larch (*Larix sibirica*) Stand of Mongolia. *Jour. Korean For. Soc.* 98, 609–617 (2009).

Parker, J.L., Fernandez, I.J., Rustad, L.E. & Norton, S.A. Effects of Nitrogen Enrichment, Wildfire, and Harvesting on Forest Soil Carbon and Nitrogen. *Soil Sci. Soc. Am. J.* 65, 1248–1255 (2001).

Parker, J.L., Fernandez, I.J., Rustad, L.E. & Norton, S.A. Soil organic matter fractions in experimental forested watersheds. *Water Air Soil Pollut.* 138, 101–121 (2002).

Pennock, D.J. & van Kessel, C. Clear-cut forest harvest impacts on soil quality indicators in the mixedwood forest of Saskatchewan, Canada. *Geoderma* 75, 13–32 (1997).

Piirainen, S., Finer, L. & Starr, M. Changes in forest floor and mineral soil carbon and nitrogen stocks in a boreal forest after clear-cutting and mechanical site preparation. *European Journal of Soil Science* (2015). doi: 10.1111/ejss.12264

Powers, M.D. *et al.* Carbon stocks across a chronosequence of thinned and unmanaged red pine (*Pinus resinosa*) stands. *Ecol. Appl.* 22, 1297–1307 (2012).

Powers, R.F. *et al.* The North American long-term soil productivity experiment: Findings from the first decade of research. *For. Ecol. Manag.* 220, 31–50 (2005).

Prest, D., Kellman, L. & Lavigne, M.B. Mineral soil carbon and nitrogen still low three decades following clearcut harvesting in a typical Acadian Forest stand. *Geoderma* 214–215, 62–69 (2014).

Richards, A.E., Dalal, R.C. & Schmidt, S. Soil carbon turnover and sequestration in native subtropical tree plantations. *Soil Biol. Biochem.* 39, 2078–2090 (2007).

Roberts, B.A., Deering, K.W. & Titus, B.D. Effects of intensive harvesting on forest floor properties in *Betula papyrifera* stands in Newfoundland. *J. Veg. Sci.* 9, 521–528 (1998).

Rothstein, D.E. & Spaulding, S.E. Replacement of wildfire by whole-tree harvesting in jack pine forests: Effects on soil fertility and tree nutrition. *For. Ecol. Manag.* 260, 1164–1174 (2010).

Rubio, A. & Escudero, A. Clear-cut effects on chestnut forest soils under stressful conditions: lengthening of time-rotation. *For. Ecol. Manag.* 183, 195–204 (2003).

Sanchez, F.G. *et al.* Soil carbon, after 3 years, under short-rotation woody crops grown under varying nutrient and water availability. *Biomass Bioenerg.* 31, 793–801 (2007).

Sanchez, F.G. *et al.* Effects of organic matter removal and soil compaction on fifth-year mineral soil carbon and nitrogen contents for sites across the United States and Canada. *Can. J. For. Res.* 36, 565–576 (2006).

Sanscrainte, C.L., Peterson, D.L. & McKay, S. Carbon Storage and Soil Properties in Late-successional and Second-growth Subalpine Forests in the North Cascade Range, Washington. *Northwest Sci.* 77, 297–307 (2003).

Schmidt, M.G., Macdonald, S.E. & Rothwell, R.L. Impacts of harvesting and mechanical site preparation on soil chemical properties of mixed-wood boreal forest sites in Alberta. *Can. J. Soil Sci.* 76, 531–540 (1996).

Schrumpf, M., Zech, W., Lehmann, J. & Lyaruu, H.V.C. TOC, TON, TOS and TOP in rainfall, throughfall, litter percolate and soil solution of a montane rainforest succession at Mt. Kilimanjaro, Tanzania. *Biogeochemistry* 78, 361–387 (2006).

Shutou, K. & Nakane, K. Change in soil carbon cycling for stand development of Japanese cedar (*Cryptomeria japonica*) plantations following clear-cutting. *Ecol. Res.* 19, 233–244 (2004).
Silver, W.L., Scatena, F.N., Johnson, A.H., Siccama, T.G. & Watt, F. At What Temporal Scales Does Disturbance Affect Belowground Nutrient Pools? *Biotropica* 28, 441–457 (1996).
Simard, D.G., Fyles, J.W., Paré, D. & Nguyen, T. Impacts of clearcut harvesting and wildfire on soil nutrient status in the Quebec boreal forest. *Can. J. Soil Sci*. 81, 229–237 (2001).
Slesak, R.A., Schoenholtz, S.H. & Harrington, T.B. Soil carbon and nutrient pools in Douglas-fir plantations 5 years after manipulating biomass and competing vegetation in the Pacific Northwest. *For. Ecol. Manag.* 262, 1722–1728 (2011).
Slesak, R.A., Schoenholtz, S.H., Harrington, T.B. & Meehan, N.A. Initial Response of Soil Carbon and Nitrogen to Harvest Intensity and Competing Vegetation Control in Douglas-Fir (*Pseudotsuga menziesii*) Plantations of the Pacific Northwest. *For. Sci.* 57, 26–35 (2011).
Smith, C.K., Coyea, M.R. & Munson, A.D. Soil carbon, nitrogen, and phosphorus stocks and dynamics under disturbed black spruce forests. *Ecol. Appl.* 10, 775–788 (2000).
Smith, N.R., Kishchuk, B.E. & Mohn, W.W. Effects of Wildfire and Harvest Disturbances on Forest Soil Bacterial Communities. *Appl. Environ. Microbiol.* 74, 216–224 (2008).
Solomon, D., Fritzsche, F., Tekalign, M., Lehmann, J. & Zech, W. Soil Organic Matter Composition in the Subhumid Ethiopian Highlands as Influenced by Deforestation and Agricultural Management. *Soil Sci. Soc. Am. J.* 66, 68–82 (2002).
Tang, J., Bolstad, P.V. & Martin, J.G. Soil carbon fluxes and stocks in a Great Lakes forest chronosequence. *Glob. Change Biol.* 15, 145–155 (2009).
Tang, X. *et al*. Different patterns of ecosystem carbon accumulation between a young and an old-growth subtropical forest in Southern China. *Plant Ecol.* 212, 1385–1395 (2011).
Taylor, A.R., Wang, J.R. & Chen, H.Y.H. Carbon storage in a chronosequence of red spruce (*Picea rubens*) forests in central Nova Scotia, Canada. *Can. J. For. Res.* 37, 2260–2269 (2007).
Trettin, C.C., Jurgensen, M.F., Gale, M.R. & McLaughlin, J.W. Recovery of carbon and nutrient pools in a northern forested wetland 11 years after harvesting and site preparation. *For. Ecol. Manag.* 262, 1826–1833 (2011).
Turner, J. & Lambert, M. Change in organic carbon in forest plantation soils in eastern Australia. *For. Ecol. Manag.* 133, 231–247 (2000).
Uhl, C., Jordan, C., Clark, K., Clark, H. & Herrera, R. Ecosystem recovery in Amazon caatinga forest after cutting, cutting and burning, and bulldozer clearing treatments. *Oikos* 38, 313–320 (1982).
Uri, V. *et al.* Biomass production and carbon sequestration in a fertile silver birch (*Betula pendula* Roth) forest chronosequence. *For. Ecol. Manag.* 267, 117–126 (2012).
Ussiri, D.A.N & Johnson, C.E. Organic matter composition and dynamics in a northern hardwood forest ecosystem 15 years after clear-cutting. *For. Ecol. Manag.* 240, 131–142 (2007).
Waldrop, M.P., McColl, J.G. & Powers, R.F. Effects of Forest Postharvest Management Practices on Enzyme Activities in Decomposing Litter. *Soil Sci. Soc. Am. J.* 67, 1250–1256 (2003).
Wei, Y. *et al.* Variation in Carbon Storage and Its Distribution by Stand Age and Forest Type in Boreal and Temperate Forests in Northeastern China. *Plos One* 8, e72201 (2013).
Xiang, W., Chai, H., Tian, D. & Peng, C. Marginal effects of silvicultural treatments on soil nutrients following harvest in a Chinese fir plantation. *Soil Sci. Plant Nutr.* 55, 523–531 (2009).
Xu, D.P., Yang, Z.J., Dell, B. & Gong, M. Effects of Site Management in *Eucalyptus urophylla* Plantations in Guangdong Province, China. In: Nambiar, E.K.S., Tiarks, A., Cossalter, C. & Ranger, J. (Eds). Site Management and Productivity in Tropical Plantation Forests: A Progress Report, Workshop proceedings, Kerala, India, Published by Center for International Forestry Research Bogor, Indonesia, pp. 31–40. (2000).
Yanai, R.D., Arthur, M.A., Siccama, T.G. & Federer, C.A. Challenges of measuring forest floor organic matter dynamics: Repeated measures from a chronosequence. *For. Ecol. Manag.* 138, 273–283 (2000).
Yarie, J. Effects of selected forest management practices on environmental parameters related to successional development on the Tanana River floodplain, interior Alaska. *Can. J. For. Res.* 23, 1001–1014 (1993).
Zabowski, D., Chambreau, D., Rotramel, N. & Thies, W.G. Long-term effects of stump removal to control root rot on forest soil bulk density, soil carbon and nitrogen content. *For. Ecol. Manag.* 255, 720–727 (2008).
Zerva, A., Ball, T., Smith, K.A. & Mencuccini, M. Soil carbon dynamics in a Sitka spruce (*Picea sitchensis* (Bong.) Carr.) chronosequence on a peaty gley. *For. Ecol. Manag.* 205, 227–240 (2005).
Zhang, Y., Mitchell, M.J., Driscoll, C.T. & Likens, G.E. Changes in soil sulfur constituents in a forested watershed 8 years after whole-tree harvesting. *Can. J. For. Res.* 29, 356–364 (1999).
Zhong, Z. & Makeschin, F. Soil biochemical and chemical changes in relation to mature spruce (*Picea abies*) forest conversion and regeneration. *J. Plant Nutr. Soil Sci.* 166, 291–299 (2003).
Zummo, L.M. & Friedland, A.J. Soil carbon release along a gradient of physical disturbance in a harvested northern hardwood forest. *For. Ecol. Manag.* 261, 1016–1026 (2011).

Effects of thinning (conventional harvest) on soil C stocks

Abella, S.R. Thinning Pine Plantations to Reestablish Oak Openings Species in Northwestern Ohio. *Environ. Manage.* 46, 391–403 (2010).

Arunachalam, A., Maithani, K., Pandey, H.N. & Tripathi, R.S. The impact of disturbance on detrital dynamics and soil microbial biomass of a *Pinus kesiya* forest in north-east India. *For. Ecol. Manag.* 88, 273–282 (1996).

Baena, C.W. *et al.* Thinning and recovery effects on soil properties in two sites of a Mediterranean forest, in Cuenca Mountain (South-eastern of Spain). *For. Ecol. Manag.* 308, 223–230 (2013).

Barbhuiya, A.R. *et al.* Dynamics of soil microbial biomass C, N and P in disturbed and undisturbed stands of a tropical wet-evergreen forest. *Eur. J. Soil Biol.* 40, 113–121 (2004).

Barbhuiya, A.R., Arunachalam, A., Pandey, H.N., Khan, M.L. & Arunachalam, K. Fine root dynamics in undisturbed and disturbed stands of a tropical wet evergreen forest in northeast India. *Trop. Ecol.* 53, 69–79 (2012).

Boerner, R.E.J., Coates, A.T., Yaussy, D.A. & Waldrop, T.A. Assessing Ecosystem Restoration Alternatives in Eastern Deciduous Forests: The View from Belowground. *Restor. Ecol.*16, 425–434 (2008).

Bravo-Oviedo, A., Ruiz-Peinado, R., Modrego, P., Alonso, R. & Montero, G. Forest thinning impact on carbon stock and soil conditions in Southern European populations of *P. sylvestris* L. *For. Ecol. Manag. 357, 259-267 (2015).*

Chatterjee, A., Vance, G.F., Pendall, E. & Stahl, P.D. Timber harvesting alters soil carbon mineralization and microbial community structure in coniferous forests. *Soil Biol. Biochem.* 40, 1901–1907 (2008).

Cheng, X. *et al*. Short-term effects of thinning on soil respiration in a pine (*Pinus tabulaeformis*) plantation. *Biol. Fertil. Soils* 50, 357–367 (2014).

Christophel, D., Höllerl, S., Prietzel, J. & Steffens, M. Long-term development of soil organic carbon and nitrogen stocks after shelterwood- and clear-cutting in a mountain forest in the Bavarian Limestone Alps. *Eur. J. Forest Res.* 134, 623–640 (2015).

Christophel, D., Spengler, S., Schmidt, B., Ewald, J. & Prietzel, J. Customary selective harvesting has considerably decreased organic carbon and nitrogen stocks in forest soils of the Bavarian Limestone Alps. *For. Ecol. Manag.* 305, 167–176 (2013).

Congdon, R.A. & Herbohn, J.L. Ecosystem Dynamics of Disturbed and Undisturbed Sites in North Queensland Wet Tropical Rain Forest. I. Floristic Composition, Climate and Soil Chemistry. *J. Trop. Ecol.* 9, 349–363 (1993).

Dannenmann, M. *et al.* The effect of forest management on trace gas exchange at the pedosphere–atmosphere interface in beech (*Fagus sylvatica* L.) forests stocking on calcareous soils. *Eur. J. Forest Res.* 126, 331–346 (2007).

Dannenmann, M., Gasche, R., Ledebuhr, A. & Papen, H. Effects of forest management on soil N cycling in beech forests stocking on calcareous soils. *Plant Soil* 287, 279–300 (2006).

Dannenmann, M., Gasche, R. & Papen, H. Nitrogen turnover and N_2O production in the forest floor of beech stands as influenced by forest management. *J. Plant Nutr. Soil Sci.* 170, 134–144 (2007).

Dore, S. *et al.* Carbon and water fluxes from ponderosa pine forests disturbed by wildfire and thinning. *Ecol. Appl.* 20, 663–683 (2010).

Fernandez, I., Álvarez-González, J.G., Carrasco, B., Ruíz-González, A.D. & Cabaneiro, A. Post-thinning soil organic matter evolution and soil CO_2 effluxes in temperate radiata pine plantations: impacts of moderate thinning regimes on the forest C cycle. *Can. J. For. Res.* 42, 1953–1964 (2012).

Ganzhorn, J.U., Ganzhorn, A.W., Abraham, J.P., Andriamanarivo, L. & Ramananjatovo, A. The impact of selective logging on forest structure and tenrec populations in western Madagascar. *Oecologia* 84, 126–133 (1990).

Giai, C. & Boerner, R.E.J. Effects of ecological restoration on microbial activity, microbial functional diversity, and soil organic matter in mixed-oak forests of southern Ohio, USA. *Appl. Soil Ecol.* 35, 281–290 (2007).

Gillon, D., Houssard, C., Valette, J.C. & Rigolot, E. Nitrogen and phosphorus cycling following prescribed burning in natural and managed Aleppo pine forests. *Can. J. For. Res.* 29, 1237–1247 (1999).

Grady, K.C. & Hart, S.C. Influences of thinning, prescribed burning, and wildfire on soil processes and properties in southwestern ponderosa pine forests: A retrospective study. *For. Ecol. Manag.* 234, 123–135 (2006).

Grüneberg, E., Schöning, I., Hessenmöller, D., Schulze, E.D. & Weisser, W.W. Organic layer and clay content control soil organic carbon stocks in density fractions of differently managed German beech forests. *For. Ecol. Manag.* 303, 1–10 (2013).

Gundale, M.J. *et al.* Restoration treatments in a Montana ponderosa pine forest: Effects on soil physical, chemical and biological properties. *For. Ecol. Manag.* 213, 25–38 (2005).

Hall, S.J. & Marchand, P.J. Effects of stand density on ecosystem properties of subalpine forests in the southern Rocky Mountains, USA. *Ann. For. Sci.* 67, 102 (2010).

Hungate, B.A., Hart, S.C., Selmants, P.C., Boyle, S.I. & Gehring, C.A. Soil responses to management, increased precipitation, and added nitrogen in ponderosa pine forests. *Ecol. Appl.* 17, 1352–1365 (2007).

Hwang, J. & Son, Y. Short-term effects of thinning and liming on forest soils of pitch pine and Japanese larch plantations in central Korea. *Ecol. Res.* 21, 671–680 (2006).

Inagaki, Y., Nakanishi, A. & Fukata, H. Soil properties and nitrogen utilization of hinoki cypress as affected by strong thinning under different climatic conditions in the Shikoku and Kinki districts in Japan. *J. For. Res.* 16, 405–413 (2011).

Inagaki, Y. *et al.* Leaf-litter nitrogen concentration in hinoki cypress forests in relation to the time of leaf-fall under different climatic conditions in Japan. *Ecol. Res.* 25, 429–438 (2010).

Ishizuka, S., Tsuruta, H. & Murdiyarso, D. An intensive field study on CO_2, CH_4, and N_2O emissions from soils at four land-use types in Sumatra, Indonesia. *Glob. Biogeochem. Cycle* 16, 1049 (2002).

Jonard, M., Misson, L. & Ponette, Q. Long-term thinning effects on the forest floor and foliar nutrient status of Norway spruce stands in Belgian Ardennes. *Can. J. For. Res.* 36, 2684–2695 (2006).

Jurgensen, M., Tarpey, R., Pickens, J., Kolka, R. & Palik, B. Long-term Effect of Silvicultural Thinnings on Soil Carbon and Nitrogen Pools. *Soil Sci. Soc. Am. J.* 76, 1418–1425 (2012).

Kaye, J.P. & Hart, S.C. Restoration and Canopy-Type Effects on Soil Respiration in a Ponderosa Pine-Bunchgrass Ecosystem. *Soil Sci. Soc. Am. J.* 62, 1062–1072 (1998).

Keller, M., Asner, G.P., Silva, N. & Palace, M. Sustainability of Selective Logging of Upland Forests in the Brazilian Amazon: Carbon Budgets and Remote Sensing as Tools for Evaluation of Logging Effects. In: Zarin, D.J., Alavalapati, J.R.R., Putz, F.E. & Schmink, M. (Eds). Working Forests in the Neotropics: Conservation through Sustainable Management? (Biology and Resource Management Series). Columbia University Press, New York (2004).

Kennard, D.K. & Gholz, H.L. Effects of high- and low-intensity fires on soil properties and plant growth in a Bolivian dry forest. *Plant Soil* 234, 119–129 (2001).

Kunhamu, T.K., Kumar, B.M. & Viswanath, S. Does thinning affect litterfall, litter decomposition, and associated nutrient release in *Acacia mangium* stands of Kerala in peninsular India? *Can. J. For. Res.* 39, 792–801 (2009).

Lasco, R.D. *et al.* Carbon stocks assessment of a selectively logged dipterocarp forest and wood processing mill in the Philippines. *J. Trop. For. Sci.* 18, 166–172 (2006).

Lee, J., Morrison, I.K., Leblanc, J.D., Dumas, M.T. & Cameron, D.A. Carbon sequestration in trees and regrowth vegetation as affected by clearcut and partial cut harvesting in a second-growth boreal mixedwood. *For. Ecol. Manag.* 169, 83–101 (2002).

Liechty, H.O., Shelton, M.G., Luckow, K.R. & Turton, D.J. Impacts of Shortleaf Pine-Hardwood Forest Management on Soils in the Ouachita Highlands: A Review. *South. J. Appl. For.* 26, 43–51 (2002).

Lindo, Z. & Visser, S. Microbial biomass, nitrogen and phosphorus mineralization, and mesofauna in boreal conifer and deciduous forest floors following partial and clear-cut harvesting. *Can. J. For. Res.* 33, 1610–1620 (2003).

Lucas-Borja, M.E. *et al.* Soil microbial community structure and activity in monospecific and mixed forest stands, under Mediterranean humid conditions. *Plant Soil* 354, 359–370 (2012).

Maasen, S. & Wirth, S. Soil microbiological monitoring of a pine forest after partial thinning for stand regeneration with beech seedlings. *Soil Sci. Plant Nutr.* 50, 815–819 (2004).

Matsuzaki, E., Sanborn, P., Fredeen, A.L., Shaw, C.H. & Hawkins, C. Carbon stocks in managed and unmanaged old-growth western redcedar and western hemlock stands of Canada's inland temperate rainforests. *For. Ecol. Manag.* 297, 108–119 (2013).

Mattson, K.G. & Smith, H.C. Detrital organic matter and soil CO_2 efflux in forests regenerating from cutting in west Virginia. *Soil Biol. Biochem.* 25, 1241–1248 (1993).

Merino, A., Real, C., Alvarez-Gonzalez, J.G. & Rodriguez-Guitian, M.A. Forest structure and C stocks in natural *Fagus sylvatica* forest in southern Europe: The effects of past management. *For. Ecol. Manag.* 250, 206–214 (2007).

Moghaddas, E.E.Y. & Stephens, S.L. Thinning, burning, and thin-burn fuel treatment effects on soil properties in a Sierra Nevada mixed-conifer forest. *For. Ecol. Manag.* 250, 156–166 (2007).

Mund, M. & Schulze, E.D. Impacts of forest management on the carbon budget of European beech (*Fagus sylvatica*) forests. *Allg. Forst- u. J.-Ztg.* 177 Jg., 3/4, 47–63 (2006).

Nakanishi, A., Inagaki, Y., Osawa, N., Shibata, S. & Hirata, K. Effects of patch cutting on leaf nitrogen nutrition in hinoki cypress (*Chamaecyparis obtusa* Endlicher) at different elevations along a slope in Japan. *J. For. Res.* 14, 388–393 (2009).

Negrete-Yankelevich, S., Fragoso, C., Newton, A.C. & Heal, O.W. Successional changes in soil, litter and macroinvertebrate parameters following selective logging in a Mexican Cloud Forest. *Appl. Soil Ecol.* 35, 340–355 (2007).

Nobles, M.M., Dillon, W. & Mbila, M. Initial Response of Soil Nutrient Pools to Prescribed Burning and Thinning in a Managed Forest Ecosystem of Northern Alabama. *Soil Sci. Soc. Am. J.* 73, 285–292 (2009).

Novak, J., Slodicak, M. & Dusek, D. Thinning effects on forest productivity and site characteristics in stands of *Pinus sylvestris* in the Czech Republic. *For. Syst.* 20, 464–474 (2011).

Olander, L.P. *et al.* Surface Soil Changes Following Selective Logging in an Eastern Amazon Forest. *Earth Interact.* 9, Paper 4, pp. 1–18 (2005).

Pérez, C.A., Carmona, M.R., Farina, J.M. & Armesto, J.J. Selective logging of lowland evergreen rainforests in Chiloé Island, Chile: Effects of changing tree species composition on soil nitrogen transformations. *For. Ecol. Manag.* 258, 1660–1668 (2009).

Powers, M., Kolka, R., Palik, B., McDonald, R. & Jurgensen, M. Long-term management impacts on carbon storage in Lake States forests. *For. Ecol. Manag.* 262, 424–431 (2011).

Powers, M.D. *et al.* Carbon stocks across a chronosequence of thinned and unmanaged red pine (*Pinus resinosa*) stands. *Ecol. Appl.* 22, 1297–1307 (2012).

Ruiz-Peinado, R., Bravo-Oviedo, A., Lopez-Senespleda, E., Montero, G. & Rio, M. Do thinnings influence biomass and soil carbon stocks in Mediterranean maritime pinewoods? *Eur. J. Forest Res.* 132, 253–262 (2013).

Ryu, S.R., Concilio, A., Chen, J., North, M. & Ma, S. Prescribed burning and mechanical thinning effects on belowground conditions and soil respiration in a mixed-conifer forest, California. *For. Ecol. Manag.* 257. 1324–1332 (2009).

Saner, P., Loh, Y.Y., Ong, R.C. & Hector, A. Carbon Stocks and Fluxes in Tropical Lowland Dipterocarp Rainforests in Sabah, Malaysian Borneo. *Plos One* 7, e29642 (2012).

Saynes, V., Etchevers, J.D., Galicia, L., Hidalgo, C. & Campo, J. Soil carbon dynamics in high-elevation temperate forests of Oaxaca (Mexico): thinning and rainfall effects. *Bosque* 33, 3–11 (2012).

Selig, M.F., Seiler, J.R. & Tyree, M.C. Soil Carbon and CO_2 Efflux as Influenced by the Thinning of Loblolly Pine (*Pinus taeda* L.) Plantations on the Piedmont of Virginia. *For. Sci.* 54, 58–66 (2008).

Skovsgaard, J.P., Stupak, I. & Vesterdal, L. Distribution of biomass and carbon in even-aged stands of Norway spruce (*Picea abies* (L.) Karst.): A case study on spacing and thinning effects in northern Denmark. *Scand. J. Forest Res.* 21, 470–488 (2006).

Slodicak, M., Novak, J. & Skovsgaard, J.P. Wood production, litter fall and humus accumulation in a Czech thinning experiment in Norway spruce (*Picea abies* (L.) Karst.). *For. Ecol. Manag.* 209, 157–166 (2005).

Sullivan, B.W. *et al.* Thinning reduces soil carbon dioxide but not methane flux from southwestern USA ponderosa pine forests. *For. Ecol. Manag.* 255, 4047–4055 (2008).

Switzer, J.M., Hope, G.D., Grayston, S.J. & Prescott, C.E. Changes in soil chemical and biological properties after thinning and prescribed fire for ecosystem restoration in a Rocky Mountain Douglas-fir forest. *For. Ecol. Manag.* 275, 1–13 (2012).

Tan, X., Chang, S.C., Comeau, P.G. & Wang, Y. Thinning Effects on Microbial Biomass, N Mineralization, and Tree Growth in a Mid-Rotation Fire-Origin Lodgepole Pine Stand in the Lower Foothills of Alberta, Canada. *For. Sci.* 54, 465–474 (2008).

Thibodeau, L., Raymond, P., Camiré, C. & Munson, A.D. Impact of precommercial thinning in balsam fir stands on soil nitrogen dynamics, microbial biomass, decomposition, and foliar nutrition. *Can. J. For. Res.* 30, 229–238 (2000).

Tian, D.L. *et al.* Effects of thinning and litter fall removal on fine root production and soil organic carbon content in Masson pine plantations. *Pedosphere* 20, 486–493 (2010).

Tiedemann, A.R., Mason, R.R. & Wickman, B.E. Forest Floor and Soil Nutrients Five Years after Urea Fertilization in a Grand Fir Forest. *Northwest Sci.* 72, 88–95 (1998).

Uri, V. *et al.* Biomass production and carbon sequestration in a fertile silver birch (*Betula pendula* Roth) forest chronosequence. *For. Ecol. Manag.* 267, 117–126 (2012).

Vargas, R., Hasselquist, N., Allen, E.B. & Allen, M.F. Effects of a Hurricane Disturbance on Aboveground Forest Structure, Arbuscular Mycorrhizae and Belowground Carbon in a Restored Tropical Forest. *Ecosystems* 13, 118–128 (2010).

Vesterdal, L., Dalsgaard, M., Felby, C., Raulund-Rasmussen, K. & Jørgensen, B.B. Effects of thinning and soil properties on accumulation of carbon, nitrogen and phosphorus in the forest floor of Norway spruce stands. *For. Ecol. Manag.* 77, 1–10 (1995).

Vijayanathan, J., Yahya, A.Z., Yaacob, A., Kassim, A.S. & Chik, S.W. Impact of Thinning of *Acacia Mangium* Plantation on Soil Chemical Properties. *Malaysian Journal of Soil Science* 15, 75–85 (2011).

Villela, D.M., Nascimento, M.T., Aragao, L.E.O.C. & Gama, D.M. Effect of selective logging on forest structure and nutrient cycling in a seasonally dry Brazilian Atlantic forest. *J. Biogeogr.* 33, 506–516 (2006).

Yuan, S.F., Ren, H., Liu, N., Wang, J. & Guo, Q.F. Can thinning of overstorey trees and planting of native tree saplings increase the establishment of native trees in exotic Acacia plantations in South China? *J. Trop. For. Sci.* 25, 79–95 (2013).

Effects of intensive harvest on soil C stocks

Bélanger, N., Paré, D. & Yamasaki, S.H. The soil acid-base status of boreal black spruce stands after whole-tree and stem-only harvesting. *Can. J. For. Res.* 33, 1874–1879 (2003).

Belleau, A., Brais, S. & Paré, D. Soil Nutrient Dynamics after Harvesting and Slash Treatments in Boreal Aspen Stands. *Soil Sci. Soc. Am. J.* 70, 1189–1199 (2006).

Brandtberg, P.O. & Olsson, B.A. Changes in the effects of whole-tree harvesting on soil chemistry during 10 years of stand development. *For. Ecol. Manag.* 277, 150–162 (2012).

Butnor, J.R., Johnsen, K.H. & Sanchez, F.G. Whole-tree and forest floor removal from a loblolly pine plantation have no effect on forest floor CO_2 efflux 10 years after harvest. *For. Ecol. Manag.* 227, 89–95 (2006).

Carter, M.C., Dean, T.J., Zhou, M., Messina, M.G. & Wang, Z. Short-term changes in soil C, N, and biota following harvesting and regeneration of loblolly pine (*Pinus taeda* L.). *For. Ecol. Manag.* 164, 67–88 (2002).

Chen, C.R. & Xu, Z.H. Soil carbon and nitrogen pools and microbial properties in a 6-year-old slash pine plantation of subtropical Australia: impacts of harvest residue management. *For. Ecol. Manag.* 206, 237–247 (2005).

Edeso, J.M., Merino, A., Gonzalez, M.J. & Marauri, P. Soil erosion under different harvesting managements in steep forestlands from northern Spain. *Land Degrad. Dev.* 10, 79–88 (1999).

Flaming, B.L. Effects of harvest intensity and organic matter removal on nitrogen availability and leaching at a high productivity coastal Douglas-fir site. Master's Thesis, University of Washington, pp. 131 (2001).

Fleming, R.L., Laporte, M.F., Hogan, G.D. & Hazlett, P.W. Effects of harvesting and soil disturbance on soil CO_2 efflux from a jack pine forest. *Can. J. For. Res.* 36, 589–600 (2006).

Gonçalves, J.L.M., Wichert, M.C.P., Gava, J.L. & Serrano, M.I.P. Soil Fertility and Growth of *Eucalyptus grandis* in Brazil under Different Residue Management Practices. In: Nambiar, E.K.S. (Ed). Site management and productivity in tropical plantation forests. Proceedings of Workshops in Piracicaba (Brazil) 22–26 November 2004 and Bogor (Indonesia) 6–9 November 2006. Bogor, Indonesia: Center for International Forestry Research (CIFOR) (2008).

Hardiyanto, E.B., Ryantoko, A. & Anshori, S. Effects of Site Management in *Acacia mangium* Plantations at PT. Musi Hutan Persada, South Sumatra, Indonesia. In: Nambiar, E.K.S., Tiarks, A., Cossalter, C. & Ranger, J. (Eds). Site Management and Productivity in Tropical Plantation Forests: A Progress Report, Workshop proceedings, Kerala, India, Published by Center for International Forestry Research Bogor, Indonesia, pp. 41–50 (2000).

Hardiyanto, E.B. & Wicaksono, A. Inter-rotation Site Management, Stand Growth and Soil Properties in *Acacia mangium* Plantations in South Sumatra, Indonesia. In: Nambiar, E.K.S. (Ed). Site management and productivity in tropical plantation forests. Proceedings of Workshops in Piracicaba (Brazil) 22–26 November 2004 and Bogor (Indonesia) 6–9 November 2006. Bogor, Indonesia: Center for International Forestry Research (CIFOR) (2008).

Harrington, T.B. & Schoenholtz, S.H. Effects of logging debris treatments on five-year development of competing vegetation and planted Douglas-fir. *Can. J. For. Res.* 40, 500–510 (2010).

Huang, Z., Clinton, P.W. & Davis, M.R. Post-harvest residue management effects on recalcitrant carbon pools and plant biomarkers within the soil heavy fraction in *Pinus radiata* plantations. *Soil Biol. Biochem.* 43, 404–412 (2011).

Huang, Z., Clinton, P.W., Davis, M.R. & Yang, Y. Impacts of plantation forest management on soil organic matter quality. *J. Soils Sediments* 11, 1309–1316 (2011).

Huang, Z. *et al.* Harvest residue management effects on tree growth and ecosystem carbon in a Chinese fir plantation in subtropical China. *Plant Soil* 364, 303–314 (2013).

Huong, V.D., Quang, L.T., Binh, N.T. & Dung, P.T. Site Management and Productivity of *Acacia auriculiformis* Plantations in South Vietnam. In: Nambiar, E.K.S. (Ed). Site management and productivity in tropical plantation forests. Proceedings of Workshops in Piracicaba (Brazil) 22–26 November 2004 and Bogor (Indonesia) 6–9 November 2006. Bogor, Indonesia: Center for International Forestry Research (CIFOR) (2008).

Johnson, D.W. *et al.* Effects of forest management on soil carbon: results of some long-term resampling studies. *Environ. Pollut.* 116, S201–S208 (2002).

Johnson, D.W. & Todd, D.E. Harvesting Effects on Long-Term Changes in Nutrient Pools of Mixed Oak Forest. *Soil Sci. Soc. Am. J.* 62, 1725–1735 (1998).

Jones, H.S., Beets, P.N., Kimberley, M.O. & Garrett, L.G. Harvest residue management and fertilisation effects on soil carbon and nitrogen in a 15-year-old *Pinus radiata* plantation forest. *For. Ecol. Manag.* 262, 339–347 (2011).

Jones, H.S., Garrett, L.G., Beets, P.N., Kimberley, M.O. & Oliver, G.R. Impacts of Harvest Residue Management on Soil Carbon Stocks in a Plantation Forest. *Soil Sci. Soc. Am. J.* 72, 1621–1627 (2008).

Kabzems, R. & Haeussler, S. Soil properties, aspen, and white spruce responses 5 years after organic matter removal and compaction treatments. *Can. J. For. Res.* 35, 2045–2055 (2005).

Kaarakka, L. *et al.* Effects of repeated whole-tree harvesting on soil properties and tree growth in a Norway spruce (*Picea abies* (L.) Karst.) stand. *For. Ecol. Manag.* 313, 180–187 (2014).

Klockow, P.A., D'Amato, A.W. & Bradford, J.B. Impacts of post-harvest slash and live-tree retention on biomass and nutrient stocks in *Populus tremuloides* Michx.-dominated forests, northern Minnesota, USA. *For. Ecol. Manag.* 291, 278–288 (2013).

Knoepp, J.D. & Swank, W.T. Forest Management Effects on Surface Soil Carbon and Nitrogen. *Soil Sci. Soc. Am. J.* 61, 928–935 (1997).

Kranabetter, J.M. & Chapman, B.K. Effects of forest soil compaction and organic matter removal on leaf litter decomposition in central British Columbia. *Can. J. Soil Sci.* 79, 543–550 (1999).

Laclau, J.P. *et al.* Organic residue mass at planting is an excellent predictor of tree growth in Eucalyptus plantations established on a sandy tropical soil. *For. Ecol. Manag.* 260, 2148–2159 (2010).

Laiho, R., Sanchez, F., Tiarks, A., Dougherty, P.M. & Trettin, C.C. Impacts of intensive forestry on early rotation trends in site carbon pools in the southeastern US. *For. Ecol. Manag.* 174, 177–189 (2003).

Li, Q., Allen, H.L. & Wilson, C.A. Nitrogen mineralization dynamics following the establishment of a loblolly pine plantation. *Can. J. For. Res.* 33, 364–374 (2003).

Mariani, L., Chang, S.X. & Kabzems, R. Effects of tree harvesting, forest floor removal, and compaction on soil microbial biomass, microbial respiration, and N availability in a boreal aspen forest in British Columbia. *Soil Biol. Biochem.* 38, 1734–1744 (2006).

Mathers, N.J. *et al.* How does residue management impact soil organic matter composition and quality under Eucalyptus globules plantations in southwestern Australia? *For. Ecol. Manag.* 179, 253–267 (2003).

Mathers, N.J. & Xu, Z. Solid-state ^{13}C NMR spectroscopy: characterization of soil organic matter under two contrasting residue management regimes in a 2-year-old pine plantation of subtropical Australia. *Geoderma* 114, 19–31 (2003).

Mattson, K.G. & Swank, W.T. Soil and detrital carbon dynamics following forest cutting in the Southern Appalachians. *Biol. Fertil. Soi*ls 7, 247–253 (1989).

Mendham, D.S., O'Connell, A.M., Grove, T.S. & Rance, S.J. Residue management effects on soil carbon and nutrient contents and growth of second rotation eucalypts. *For. Ecol. Manag.* 181, 357–372 (2003).

Mendham, D.S., Sankaran, K.V., O'Connell, A.M. & Grove, T.S. *Eucalyptus globulus* harvest residue management effects on soil carbon and microbial biomass at 1 and 5 years after plantation establishment. *Soil Biol. Biochem.* 34, 1903–1912 (2002).

Merino, A. & Edeso, J.M. Soil fertility rehabilitation in young *Pinus radiata* D. Don. Plantations from northern Spain after intensive site preparation. *For. Ecol. Manag.* 116, 83–91 (1999).

Morris, D.M., Kwiaton, M.M. & Duckert, D.R. Black spruce growth response to varying levels of biomass harvest intensity across a range of soil types: 15-year results. *Can. J. For. Res.* 44, 313–325 (2014).

Nzila, J.D., Bouillet, J.P., Laclau, J.P. & Ranger, J. The effects of slash management on nutrient cycling and tree growth in Eucalyptus plantations in the Congo. *For. Ecol. Manag.* 171, 209–221 (2002).

Olsson, B.A., Staaf, H., Lundkvist, H., Bengtsson, J. & Rosen, K. Carbon and nitrogen in coniferous forest soils after clear-felling and harvests of different intensity. *For. Ecol. Manag.* 82, 19–32 (1996).

Piatek, K.B. & Allen, H.L. Nitrogen Mineralization in a Pine Plantation Fifteen Years After Harvesting and Site Preparation. *Soil Sci. Soc. Am. J.* 63, 990–998 (1999).

Powers, R.F. *et al.* The North American long-term soil productivity experiment: Findings from the first decade of research. *For. Ecol. Manag.* 220, 31–50 (2005).

Rittenhouse, T.A.G., MacFarland, D.M., Martin, K.J. & Van Deelen, T.R. Downed wood associated with roundwood harvest, whole-tree harvest, and unharvested stands of aspen in Wisconsin. *For. Ecol. Manag.* 266, 239–245 (2012).

Roberts, B.A., Deering, K.W. & Titus, B.D. Effects of intensive harvesting on forest floor properties in *Betula papyrifera* stands in Newfoundland. *J. Veg. Sci.* 9, 521–528 (1998).

Rosenberg, O. & Jacobson, S. Effects of repeated slash removal in thinned stands on soil chemistry and understorey vegetation. *Silva. Fenn.* 38, 133–142 (2004).

Ross, D.J., Sparling, G.P., Burke, C.M. & Smith, C.T. Microbial biomass C and N, and mineralizable-N, in litter and mineral soil under *Pinus radiata* on a coastal sand: Influence of stand age and harvest management. *Plant Soil* 175, 167–177 (1995).

Saarsalmi, A., Tamminen, P., Kukkola, M. & Hautajärvi, R. Whole-tree harvesting at clear-felling: Impact on soil chemistry, needle nutrient concentrations and growth of Scots pine. *Scand. J. Forest Res.* 25, 148–156 (2010).

Sanchez, F.G. *et al.* Effects of organic matter removal and soil compaction on fifth-year mineral soil carbon and nitrogen contents for sites across the United States and Canada. *Can. J. For. Res.* 36, 565–576 (2006).

Shaohui, F. *et al.* Effects of Site Management in Chinese Fir (*Cunninghamia lanceolata*) Plantations in Fujian Province, China. In: Nambiar, E.K.S., Tiarks, A., Cossalter, C. & Ranger, J. (Eds). Site Management and Productivity in Tropical Plantation Forests: A Progress Report, Workshop proceedings, Kerala, India, Published by Center for International Forestry Research Bogor, Indonesia, pp. 83–86 (2000).

Siregar, S.T.H., Nurwahyudi, & Mulawarman. Effects of Inter-rotation Management on Site Productivity of *Acacia mangium* in Riau Province, Sumatra, Indonesia. In: Nambiar, E.K.S. (Ed). Site management and productivity in tropical plantation forests. Proceedings of Workshops in Piracicaba (Brazil) 22–26 November 2004 and Bogor (Indonesia) 6–9 November 2006. Bogor, Indonesia: Center for International Forestry Research (CIFOR) (2008).

Slesak, R.A., Schoenholtz, S.H. & Harrington, T.B. Soil carbon and nutrient pools in Douglas-fir plantations 5 years after manipulating biomass and competing vegetation in the Pacific Northwest. *For. Ecol. Manag.* 262, 1722–1728 (2011).

Slesak, R.A., Schoenholtz, S.H., Harrington, T.B. & Meehan, N.A. Initial Response of Soil Carbon and Nitrogen to Harvest Intensity and Competing Vegetation Control in Douglas-Fir (*Pseudotsuga menziesii*) Plantations of the Pacific Northwest. *For. Sci.* 57, 26–35 (2011).

Smaill, S.J., Clinton, P.W. & Greenfield, L.G. Postharvest organic matter removal effects on FH layer and mineral soil characteristics in four New Zealand *Pinus radiata* plantations. *For. Ecol. Manag*. 256, 558–563 (2008).

Smith, C.T. *et al.* Response of radiata pine forests to residue management and fertilisation across a fertility gradient in New Zealand. *For. Ecol. Manag.* 138, 203–223 (2000).

Smolander, A., Kitunen, V., Tamminen, P. & Kukkola, M. Removal of logging residue in Norway spruce thinning stands: Long-term changes in organic layer properties. *Soil Biol. Biochem.* 42, 1222–1228 (2010).

Smolander, A., Levula, T. & Kitunen, V. Response of litter decomposition and soil C and N transformations in a Norway spruce thinning stand to removal of logging residue. *For. Ecol. Manag.* 256, 1080–1086 (2008).

Strömgren, M., Egnell, G. & Olsson, B.A. Carbon stocks in four forest stands in Sweden 25 years after harvesting of slash and stumps. *For. Ecol. Manag.* 290, 59–66 (2013).

Tamminen, P., Saarsalmi, A., Smolander, A., Kukkola, M. & Helmisaari, H.S. Effects of logging residue harvest in thinnings on amounts of soil carbon and nutrients in Scots pine and Norway spruce stands. *For. Ecol. Manag.* 263, 31–38 (2012).

Tan, X., Chang, S.X. & Kabzems, R. Effects of soil compaction and forest floor removal on soil microbial properties and N transformations in a boreal forest long-term soil productivity study. *For. Ecol. Manag.* 217, 158–170 (2005).

Tan, X., Chang, S.X. & Kabzems, R. Soil compaction and forest floor removal reduced microbial biomass and enzyme activities in a boreal aspen forest soil. *Biol. Fertil. Soils* 44, 471–479 (2008).

Thiffault, E., Bélanger, N., Paré, D. & Munson, A.D. How do forest harvesting methods compare with wildfire? A case study of soil chemistry and tree nutrition in the boreal forest *Can. J. For. Res.* 37, 1658–1668 (2007).

Thiffault, E. *et al.* Chemical composition of forest floor and consequences for nutrient availability after wildfire and harvesting in the boreal forest. *Plant Soil* 308, 37–53 (2008).

Thiffault, E., Paré, D., Bélanger, N., Munson, A. & Marquis, F. Harvesting intensity at clear-felling in the boreal forest: impact on soil and foliar nutrient status. *Soil Sci. Soc. Am. J.* 70, 691–701 (2006).

Tutua, S.S., Xu, Z.H., Blumfield, T.J. & Bubb, K.A. Long-term impacts of harvest residue management on nutrition, growth and productivity of an exotic pine plantation of sub-tropical Australia. *For. Ecol. Manag.* 256, 741–748 (2008).

Vanguelova, E., Pitman, R., Luiro, J. & Helmisaari, H.S. Long term effects of whole tree harvesting on soil carbon and nutrient sustainability in the UK. *Biogeochemistry* 101, 43–59 (2010).

Wall, A. Effect of removal of logging residue on nutrient leaching and nutrient pools in the soil after clearcutting in a Norway spruce stand. *For. Ecol. Manag.* 256, 1372–1383 (2008).

Walmsley, J.D., Jones, D.L., Reynolds, B., Price, M.H. & Healey, J.R. Whole tree harvesting can reduce second rotation forest productivity. *For. Ecol. Manag.* 257, 1104–1111 (2009).

Wei, X., Kimmins, J.P., Peel, K. & Steen, O. Mass and nutrients in woody debris in harvested and wildfire-killed lodgepole pine forests in the central interior of British Columbia. *Can. J. For. Res.* 27, 148–155 (1997).

Wei, X., Li, Q., Waterhouse, M.J. & Armleder, H.M. Organic Matter Loading Affects Lodgepole Pine Seedling Growth. *Environ. Manage.* 49, 1143–1149 (2012).

Xu, D.P., Yang, Z.J., Dell, B. & Gong, M. Effects of Site Management in *Eucalyptus urophylla* Plantations in Guangdong Province, China. In: Nambiar, E.K.S., Tiarks, A., Cossalter, C. & Ranger, J. (Eds). Site Management and Productivity in Tropical Plantation Forests: A Progress Report, Workshop proceedings, Kerala, India, Published by Center for International Forestry Research Bogor, Indonesia, pp. 31–40 (2000).

Zabowski, D., Chambreau, D., Rotramel, N. & Thies, W.G. Long-term effects of stump removal to control root rot on forest soil bulk density, soil carbon and nitrogen content. *For. Ecol. Manag.* 255, 720–727 (2008).

Zabowski, D., Java, B., Scherer, G., Everett, R.L. & Ottmar, R. Timber harvesting residue treatment: Part 1. Responses of conifer seedlings, soils and microclimate. *For. Ecol. Manag.* 126, 25–34 (2000).
